# Photoinduced Tautomerisation of ESIPT-Capable Iridium(III) Complexes with Rationally Designed Acyclic Diaminocarbene Ligands

**DOI:** 10.1021/acs.inorgchem.5c04206

**Published:** 2026-01-13

**Authors:** Polina O. Skripnyak, Maria V. Kashina, Anzhelika A. Eremina, Sergei V. Tatarin, Stanislav I. Bezzubov, Konstantin V. Luzyanin, Mikhail A. Kinzhalov

**Affiliations:** † 48544 St Petersburg State University, 7−9 Universitetskaya Nab., Saint Petersburg 199034, Russian Federation; ‡ Kurnakov Institute of General and Inorganic Chemistry, Russian Academy of Sciences, Leninskii Prosp. 31, 119991 Moscow, Russian Federation; § Department of Chemistry, 4591 University of Liverpool, Crown Street, Liverpool L69 7ZD, United Kingdom

## Abstract

A series of ESIPT-capable Ir^III^-(acyclic diaminocarbene species) (ESIPT = Excited-state intramolecular proton transfer) exhibiting strong photoluminescence properties is described. The emission profile is strongly influenced by the nature of the azaheterocyclic fragment in the diaminocarbene ligand: pyrazine-derived species display phosphorescence bands red-shifted by approximately 100 nm compared to their pyridine analogues. This redshift is attributed to the luminescence of tautomerized species formed via an ESIPT process, wherein the iridium center enhances the basicity of the pyrazine ring, facilitating proton transfer from the C_carbene_–NH groups. This interpretation is supported by the solvatochromic emission behavior of complexes prepared and corroborated by density functional theory calculations. Prepared Ir^III^-(acyclic diaminocarbene species) complexes represent the first example of metal–organic luminophores in which the ESIPT mechanism involves direct participation of the metal center, resulting in orange emission.

## Graphical abstract

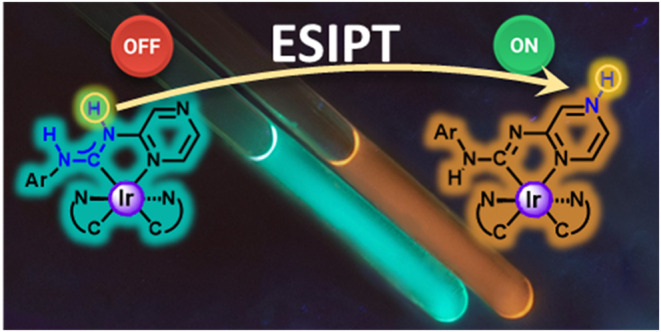

## Introduction

Excited-state intramolecular proton transfer (ESIPT), a process in which a molecule undergoes tautomerisation upon photoexcitation, plays a crucial role in various natural luminophores,^1–3^ and is used for developing advanced sensors, organic light-emitting devices, and molecular switches.^4–6^ ESIPT luminophores exhibit an exceptionally large Stokes shift compared to conventional luminophores, effectively minimizing unwanted self-reabsorption and inner-filter effects.^7^ Furthermore, the sensitivity of ESIPT mechanisms to such factors as pH, temperature, and solvent allows for precise tuning of luminescence sensors.^8^

Organometallic luminescent complexes, known for their long-lived emission and lower susceptibility to photodegradation comparing to organic luminophores, are key for innovative OLEDs and other photonic applications. These complexes may further benefit from ESIPT-involving strategies, offering potential to be better molecular probes for biological studies and improved photocatalytic performance with emission intensity influenced by the external hydrogen bonding.^9^ Only a limited number of metal–organic ESIPT luminophores have been designed, which includes the coordination of organic ligands with ESIPT potential via an intramolecular hydrogen bonding between proton-donating and proton-accepting groups (representative molecular structures of Ir^III^ complexes, which exhibited ESIPT tautomerism via intramolecular H-bond give in Figure 1).^10,11^ In rare reported cases, proton transfer can occur over a long distance, facilitated by solvent interactions or concentration gradients.^12^ The resulting tautomers typically exhibit changed from blue to green emission due to the higher efficiency of emissive metal-to-ligand charge transfer (MLCT). To the best of our knowledge, no examples of metal complexes have been reported that demonstrate ESIPT processes involving direct intercalation with metal orbitals.

**Figure 1:**
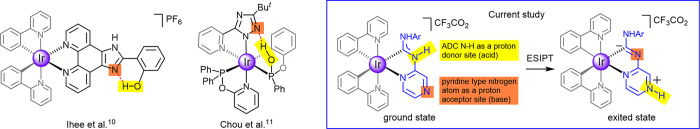
Left: Representative molecular structures of Ir^III^ complexes, which exhibited ESIPT tautomerism via an intramolecular hydrogen bond.^10,11^ Right: Strategic design of ESIPT-prone Ir^III^ acyclic diaminocarbene complexes.

Among metal complexes, Ir^III^ species have gathered considerable attention due to their synthetic adaptability, tunable photophysical characteristics, and remarkable thermal and photochemical stability.^11,13–19^ We have selected the iridium center as a strategic platform for the development of novel ESIPT luminophores as their complexes featuring acyclic diaminocarbene (ADC) ligands are distinguished by their high photoluminescence quantum yields and ability to participate in outer-sphere electron-transfer processes.^20^ We have, therefore, designed Ir^III^-(ADC) complexes featuring spatially separated proton-donor and proton-acceptor centers through the strategic incorporation of an additional nitrogen atom and uncovered that they are ESIPT-capable (Figure 1).

## Results and Discussion

### Synthesis and Characterization

A direct synthetic approach to Ir^III^ complexes with ADC ligands involves nucleophilic addition to metal-bound isocyanides. This isocyanide-based method is atom-efficient generating NH centers in the aminocarbene moiety, which can later be deprotonated.^21^ ESIPT-capable Ir^III^-(ADC) species **3**–**4** were prepared by reaction of isocyanides in [IrCl­(*C*,*N*-ppy)_2_(CNAr)] (**1**–**2**; Ar = C_6_H_4_-4-Cl, C_6_H_4_-3-CF_3_; ppy = 2-phenylpyridine) with 2-aminopyrazine in the presence of CF_3_CO_2_Ag as chloride sequestrant (Scheme 1).

**Scheme 1:**
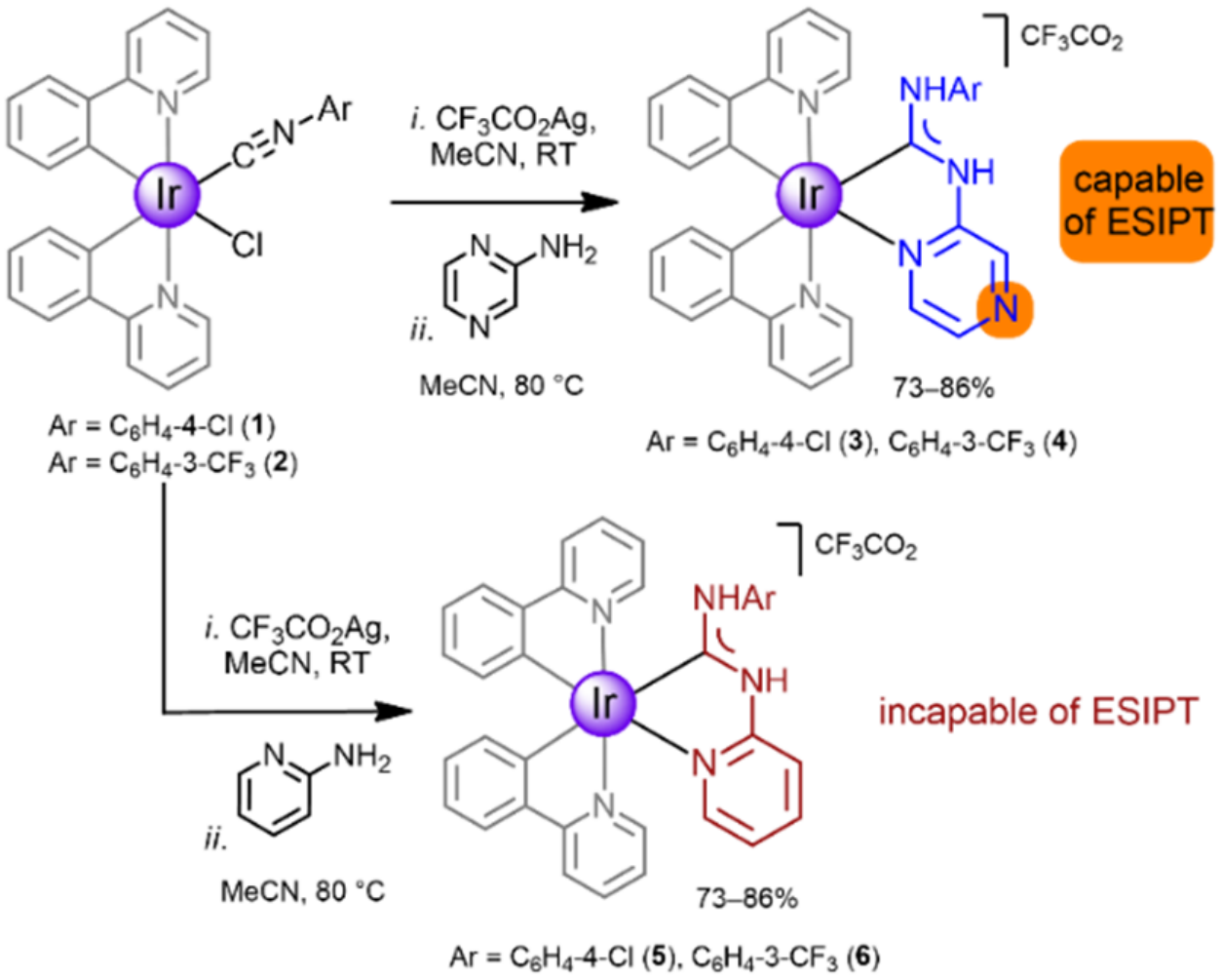
Synthetic Routes to ESIPT–capable Ir^III^-(ADC) Complexes

The coupling proceeds via the reaction of the C≡N group of the metal-bound isocyanide and the NH_2_ group of the incoming α-aminoazaheterocycle. The endocyclic *N*-atom of the α-aminoazaheterocycle chelates the metal forming a five-membered ring. Additionally, we prepared a series of ESIPT-incapable complexes **5**–**6** by replacing 2-aminopyrazine with 2-aminopyridine, allowing for a more comprehensive investigation of the proton transfer mechanism and to elucidate the critical factors governing this process. All **3**–**6** were characterized by various analytical spectroscopic and nonspectroscopic techniques (Sections S2, S6–S8 in ESI).

The diaminocarbene fragment in **3**–**6** features significant delocalization of electron density and acidity as evidenced by emergence of proton resonances for C_carbene_–NH in 14–15 ppm range.^13^ The cross-peaks in ^1^H,^1^H-NOESY spectra of **3**–**6** reveal through-space interactions among the C_carbene_–NH protons, indicating the formation of the ADC ligand where both NH groups are in *cis* configuration (Figure S1). According to the X-ray diffraction (XRD) data for the crystal structure of **4** (Figure 2), the Ir^III^ center exhibits a slightly distorted octahedral coordination environment, in which the pyrazine ring forms a CN-chelating system with the diaminocarbene fragment. Both NH groups form N–H···O H-bonds with the triflate anion (d­(H···O) = 1.919(2)–1.953(2) Å less than sum of vdW radii;^22^ ∠(N–H···Cl) = 164.9(3)° close to linear), which is consistent with the downfield C_carbene_–NH signals in NMR experimental results.

**Figure 2:**
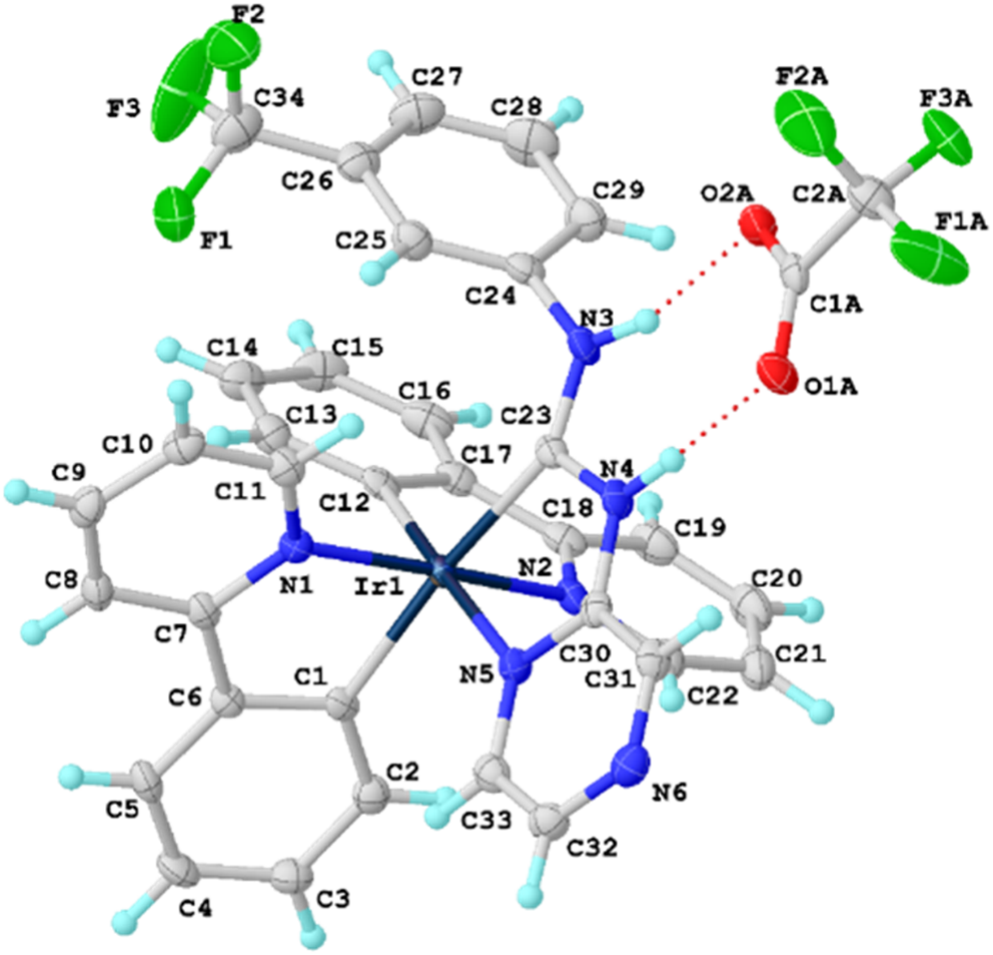
View of **4** with the atomic numbering schemes. Hydrogen labels were omitted for simplicity. The crystal data, and selected bond lengths and angles are given in the ESI, section S4.

### UV–Vis Absorption and Emission Properties

The absorption spectra of compounds **3**–**6** in CH_2_Cl_2_ or MeCN are nearly identical in the region below 320 nm, displaying intense bands [ε = (1.48–6.20) × 10^4^ M^–1^ cm^–1^], which correspond to spin-allowed π–π* ligand-centered transitions (^1^LC) (Figures 3 and S2). The main spectral differences are observed in the 350–450 nm region [ε = (2.2–18.0) × 10^3^ M^–1^ cm^–1^], where pyridine-containing complexes **5**–**6** display mixed metal-to-ligand charge transfer (MLCT) and ligand-to-ligand charge transfer (LLCT), involving the iridium center and ppy ligands. This is confirmed by TD-DFT analyses of **5**, involving FMO and NTO methods (Tables S6–S9, and Figures 4, S21–S23). In contrast, the pyrazine analogues **3**–**4** show distinct long-wavelength absorption features, with MLCT/LLCT transitions that localize the orbital density primarily on the ADC ligand, especially on the pyrazine group. This significant charge separation enhances the basicity of the pyrazine nitrogens in the excited state, creating favorable conditions for efficient proton transfer.

**Figure 3:**
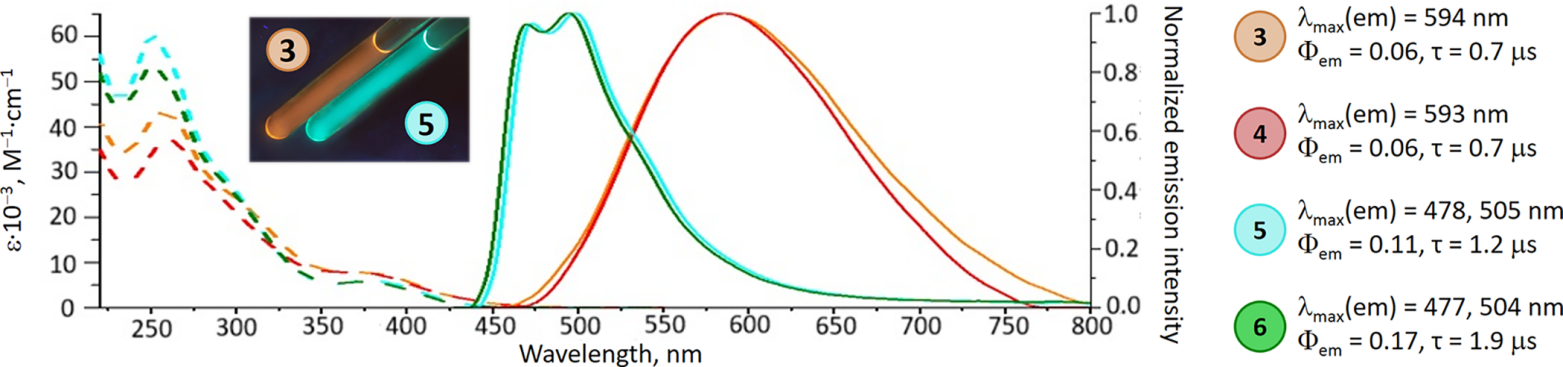
UV/vis absorption spectra and emission spectra of **3**–**6** in degassed CH_2_Cl_2_ at RT.

**Figure 4:**
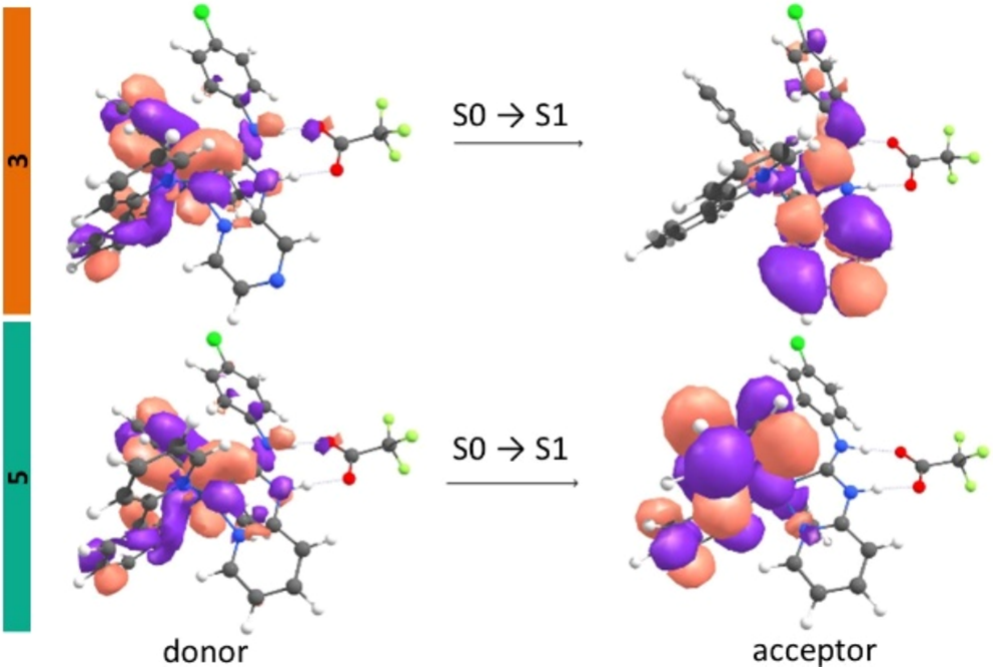
Natural transition orbitals (NTOs) representing the lowest energy transitions for **3** (top) and **5** (bottom).

Under UV excitation (365 nm), pyridine-containing compounds **5–6** exhibit intense green-blue emission in 450–650 nm range with a notable vibronic progression. This behavior is observed in both solutions (CH_2_Cl_2_ or MeCN) and in PMMA films or solid state, with quantum yields of Φ_em_ = 0.11–0.17 (Figures 3 and S6–S8). The large oxygen sensitive long-lived luminescence points to a phosphorescence nature of emission.^23^ These emission properties align with those demonstrated by other Ir^III^-*bis*-2-phenylpyridinato complexes, where the ppy ligands play a key role in emissive behavior.^24–26^

In sharp contrast, pyrazine complexes **3**–**4** show broad structureless emission bands red-shifted compared to those of **5**–**6**. In aprotic solvents (MeCN, CH_2_Cl_2_, THF), **3** demonstrates a redshift in its emission spectrum (positive solvatochromic effect: 555 nm in THF, 593 nm in CH_2_Cl_2_ and 632 nm in MeCN), attributed to the stabilization of a more polar excited state (Figure 5). The emission spectra of **3** in protic solvent (EtOH), display structured bands (λ_max_ = 458 and 487 nm, Figure 5), resembling the emission profiles of **5**–**6**. This behavior provides evidence of substantial charge transfer occurring upon photoexcitation, highlighting the influence of solvent polarity on the photophysical properties of the system. The formation of hydrogen bonds between **3** and EtOH blocks the proton-acceptor center and inhibits the ESIPT process.^27^ In the crystalline state, both complexes **3** and **5** exhibit emission spectra devoid of the long-wavelength feature associated with ESIPT product emission (Figure S8).

**Figure 5:**
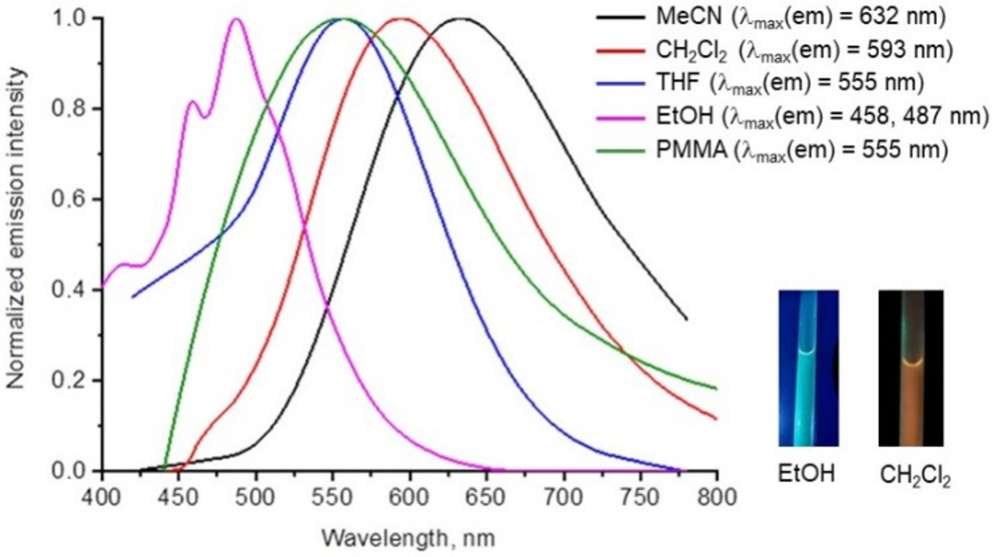
Emission spectra of **3** in different solvents and in PMMA films.

The luminescence quantum yields of **3**–**4** in solution (Φ_em_ = 6%) and PMMA films (Φ_em_ = 6%) are 2–3 times lower than those of the pyridine analogues **5**–**6** (Φ_em_ = 11–17% in CH_2_Cl_2_ and Φ_em_ = 21–26% in PMMA), indicating a significant alteration in the nature of the emissive excited state. The observed large Stokes shift, combined with evidence of intramolecular hydrogen-involved interactions, supports the manifestation of ESIPT process.^28^

### Theoretical Investigations of the Emission Behavior

To elucidate the emission behavior of **3**–**6**, theoretical calculations were conducted with respect of phosphorescent triplet states. The geometry of tautomer **3** ^**T**^ (Figure 6) was also optimized to examine the ESIPT mechanism, which entails the transfer of one NH proton from the ADC ligand to the nitrogen atom of the pyrazine moiety. The formation of this tautomer is predicted based on the acidity of the C_carbene_–NH groups and the enhanced basicity of the pyrazine ring, a consequence of electron redistribution following light absorption.

**Figure 6:**
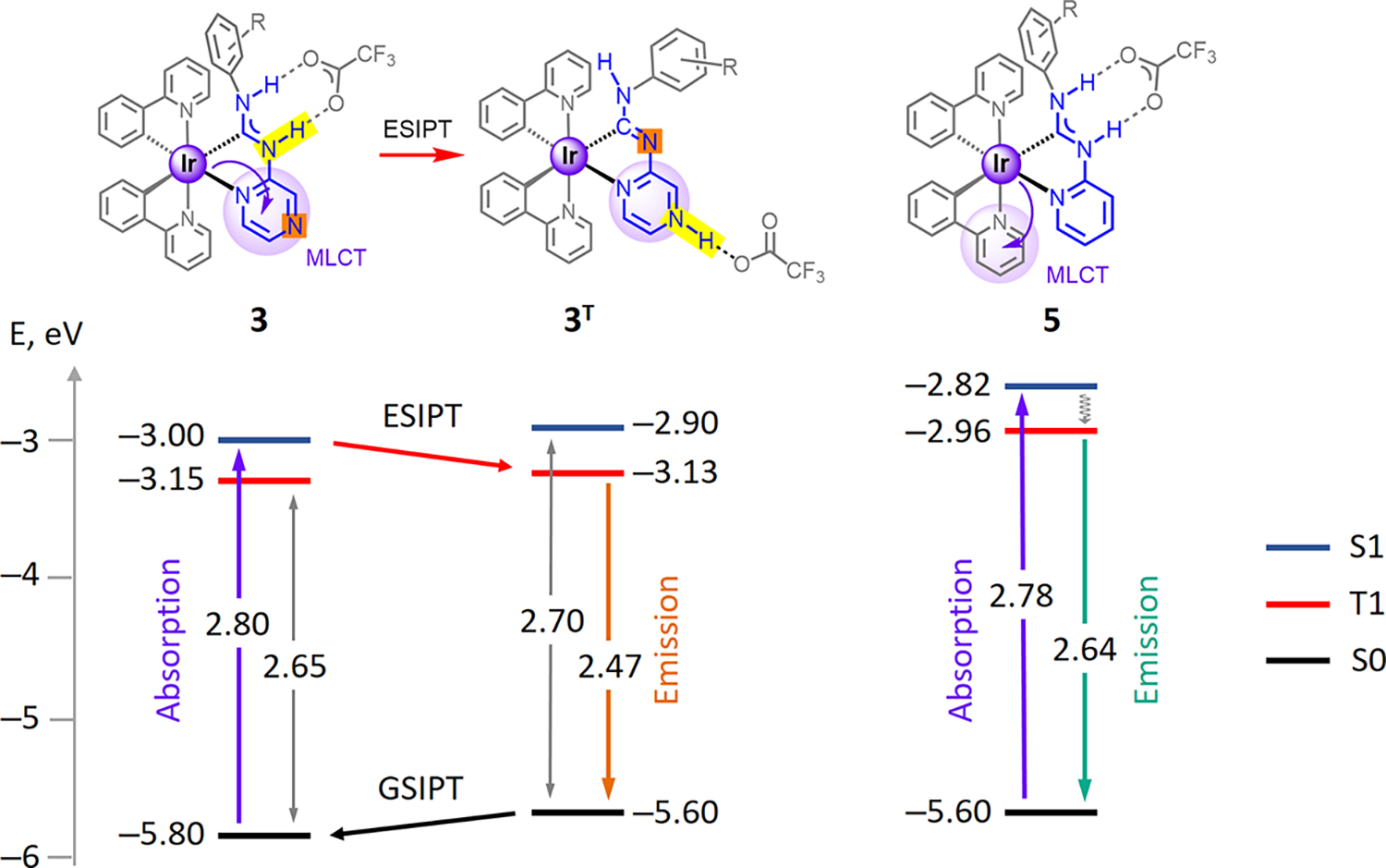
TD-DFT calculated lowest energy transitions in the structures of **3**, **3** ^**T**^ and **5** and the proposed emission mechanisms.

In the ground state (S0), the structure **3** ^**T**^ is less stable than **3** by 0.2 eV (4.6 kcal/mol, Figure 6), indicating that tautomer **3** is predominant in solution prior to excitation, with **3** ^**T**^ constituting less than 0.01% of the population. This prediction aligns with the experimental data obtained from ^1^H,^1^H-NOESY and X-ray analyses (*vide supra*). According to TD-DFT calculations, upon vertical S0→S1 excitation, the singlet excited state S1 of **3** remains energetically more favorable than that of **3** ^**T**^ by 0.1 eV (2.3 kcal/mol), which may account for the absence of additional absorption bands in the spectra of compounds **3**–**4**.

While, according to TDDFT calculations, the emissive triplet state (T1) of the predicted tautomer **3** ^**T**^ is similar in energy to that of **3**, the electron density of the lowest-energy excitation in **3** moves to the pyrazine ring (Figure 4), facilitating ESIPT (**3**→**3** ^**T**^). Furthermore, in the triplet excited state of **3** ^**T**^, the spin density is predominantly localized on the ADC ligand (ca. 81%, Figure 7), in contrary to the triplet excited state of **3**, where it is localized on the ppy ligand (ca. 65–70%) and the iridium *d* orbitals (ca. 22–23%). The T1→S0 TD-DFT derived transition energy, corresponding to the phosphorescence energy, is 2.47 eV for **3** ^**T**^ vs 2.65 eV for **3**, which translates to theoretical emission maxima at 502 and 468 nm, respectively, reflecting the experimentally observed bathochromic shift and providing strong theoretical support for the proposed ESIPT mechanism. The relaxation of **3** ^**T**^ to **3** occurs via a ground-state intramolecular proton transfer (GSIPT) reaction. For the pyridine-containing **5**, an ESIPT-non prone species, the calculated T1→S0 transition energy of 2.64 eV (corresponding to an emission wavelength of 469 nm) aligns well with the experimentally observed emission maximum, thereby validating the accuracy of the theoretical model.

**Figure 7:**
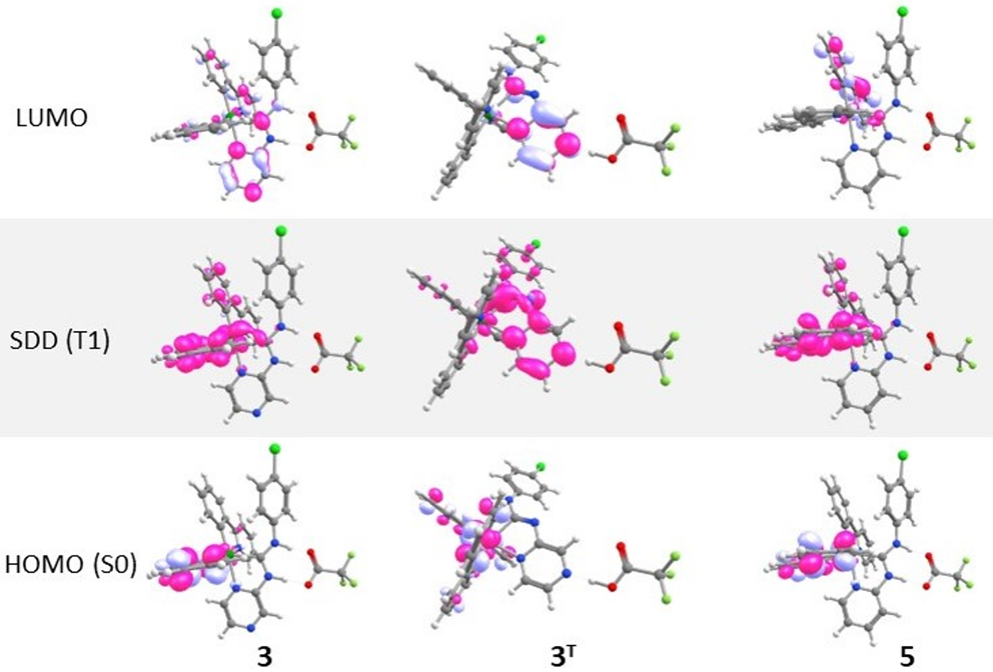
Surface plots of HOMO, LUMO molecular orbitals and spin density distribution in the lowest triplet excited state (SDD (T1)).

The proposed mechanism involves a three-step sequence. Initially, photoexcitation triggers an ultrafast proton transfer from the N–H group to the oxygen atom of a hydrogen-bonded trifluoroacetate counterion, a process analogous to known organic systems with a characteristic femtosecond-to-picosecond time scale. Subsequently, migration of the trifluoroacetate acid occurs on a slower, nanosecond time scale.^29,30^ The final step involves protonation of the *N*-donor center, yielding hydrogen-bonded ion pairs comprising the protonated complex and the trifluoroacetate anion. The cumulative timeline for this cascade is shorter than the observed emission lifetime of approximately 700 ns. This kinetic behavior supports the feasibility of our mechanism providing also an explanation for the observed large Stokes shift.

To investigate the potential migration of trifluoroacetic acid (TFA) between the two nitrogen-donor sites, we undertook the computational evaluation of the Gibbs free energy (*G*) required for the TFA dissociation and association at both the carbene and pyrazine moieties (Figure 8). Our calculations demonstrate that the initial TFA coordination is energetically favored at the diaminocarbene nitrogen over the pyrazine nitrogen by 3 kcal/mol. This computational prediction is in agreement with experimental observations from SCXRD analysis and corroborated by solution NMR spectroscopy. Although the first binding event exhibits a clear preference for the diaminocarbene site, the subsequent coordination of a second TFA molecule to the pyrazine nitrogen remains thermodynamically accessible, as evidenced by a favorable binding energy of −13 kcal/mol.

**Figure 8:**
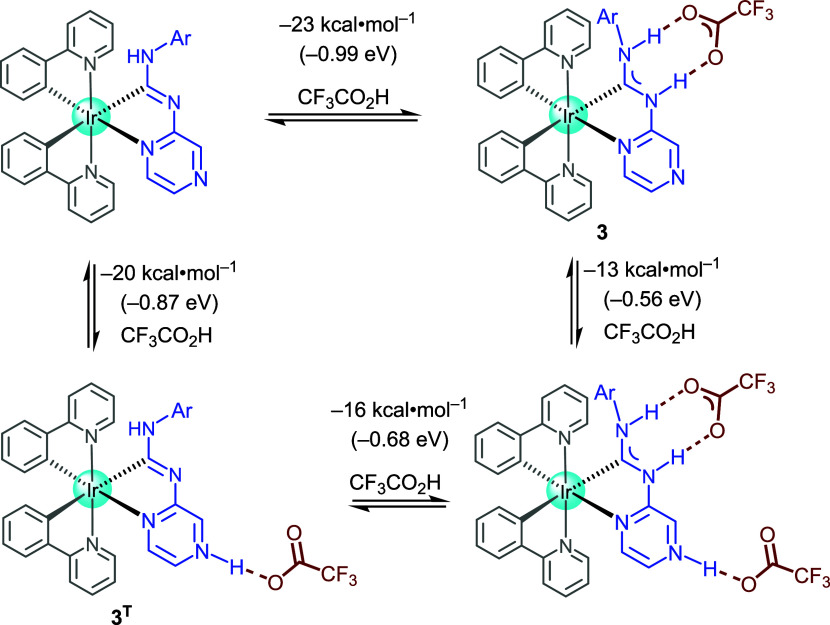
Relative energies (Δ*G* ^0^) for different binding sites and numbers of TFA molecules on complex **3**.

Given the equimolar ratio of iridium complexes and TFA in solution, the formation of a hypothetical **3** ^**T**^ structure becomes plausible through either an association–dissociation or dissociation-association pathway. The associated energy differences between these states fall within the range of typical light-induced reorganization energies observed in electron transfer within artificial molecular systems,^31^ further favoring an ESIPT mechanism.

Spectroscopic analysis of the deuterated analogue of complex **3** revealed the complete absence of the long-wavelength emission band assigned to the ESIPT product (Figure S9). This pronounced kinetic isotope effect provides definitive evidence that proton transfer is the pivotal mechanistic step in the photocycle.

## Conclusions

We report herein on the preparation of ESIPT-capable Ir^III^ acyclic diaminocarbene complexes **3**–**6**, which exhibit notable photoluminescence properties. The emission behavior is strongly influenced by the nature of the azaheterocyclic fragment: in aprotic solvents, pyrazine-containing **3**–**4** display phosphorescence bands red-shifted by ca. 100 nm compared to their pyridine analogues **5**–**6**. This redshift is attributed to the luminescence of tautomerized species formed via an ESIPT process, wherein the iridium center enhances the basicity of the pyrazine ring, facilitating proton transfer from the C_carbene_–NH groups. This interpretation is supported by the solvatochromic emission behavior of **3**–**4** and corroborated by DFT calculations. The synthesized Ir^III^-ADC complexes represent the first examples of metal–organic luminophores in which the ESIPT mechanism involves direct participation of the metal center, resulting in orange emission. The observed proton transfer between nonadjacent donor and acceptor sites within a transition metal complex is unprecedented, expanding the scope of ESIPT processes in phosphorescent metal-based systems. These findings offer significant potential for advancing fundamental understanding and practical applications in luminescence control and, potentially, photocatalysis mediated by hydrogen bonding interactions.

## Experimental Section

### Materials and Instrumentation

For details see Section S1 of the Supporting Information.

### Synthetic Work

#### Synthesis of **3**–**6**

A mixture of isocyanide complex **1** or **2** (0.02 mmol) and CF_3_CO_2_Ag (4 mg, 0.02 mmol) was suspended in MeCN (3 mL) at RT. The mixture was stirred for 5 h to give yellow solution over the colorless precipitate of AgCl, which was separated by centrifugation. The 2-aminopyridine or 2-aminopyrazine (0.02 mmol) dissolved in MeCN (2 mL) was added to centrifugate. The reaction mixture was stirred at 80 °C for 24 h to form a yellow solution, which was evaporated to dryness in vacuo at 20–25 °C. The product was purified by column chromatography on basic aluminum oxide using CH_2_Cl_2_ as eluent, reprecipitated from pentane and dried in air at room temperature.

**3**. Yield 14 mg (80%), yellow solid. HRESI^+^-MS, *m*/*z*: calcd for C_33_H_25_ClIrN_6_ 733.1453, found 733.1476 [M + H]^+^. IR (KBr, cm^–1^): 192 (low, wide), ν (N–H), 1664 (m), ν­(C=N). ^1^H NMR (CDCl_3_, δ): 5.67 (d, *J*_H,H_ = 7.6 Hz, 1H, H_ppy_), 6.05 (d, *J*_H,H_ = 7.3 Hz, 1H, H_ppy_), 6.42 (t, *J*_H,H_ = 7.3 Hz, 1H, H_ppy_), 6.46 (d, *J*_H,H_ = 8.5 Hz, 2H, H_ADC_), 6.62 (d, *J*_H,H_ = 8.5 Hz, 2H, H_ADC_), 6.75 (t, *J*_H,H_ = 7.2 Hz, 1H, H_ppy_), 6.90 (t, *J*_H,H_ = 7.3 Hz, 1H, H_ppy_), 6.98 (t, *J*_H,H_ = 7.6 Hz, 1H, H_ppy_), 7.03 (t, *J*_H,H_ = 6.5 Hz, 1H, H_ppy_), 7.17 (t, *J*_H,H_ = 7.3 Hz, 1H, H_ppy_), 7.28–7.33 (m, 2H, H_ppy_), 7.54 (d, *J*_H,H_ = 5.8 Hz, 1H, H_ppy_), 7.58 (d, *J*_H,H_ = 7.8 Hz, 1H, H_ppy_), 7.78–7.86 (m, 4H, H_ppy_), 8.06 (d, *J*_H,H_ = 3.4 Hz, 1H, H_ADC_), 8.65 (d, *J*_H,H_ = 5.6 Hz, 1H, H_ADC_), 8.99 (d, *J*_H,H_ = 1.2 Hz, 1H, H_ADC_), 13.01 (s, 1H, N–H), 14.74­(s, 1H, N–H). ^13^C­{^1^H} NMR (CDCl_3_, δ): 119.46, 119.96, 120.25, 122.13, 123.13, 123.88, 124.27, 124.57, 127.05, 128.25, 129.66, 130.10, 130.52, 130.74, 130.95, 132.84, 135.24, 136.14, 136.97, 137.88, 139.19, 140.43, 141.43, 143.65, 148.54, 151.73, 155.53, 163.09, 167.93, 168.83, 207.92 (C^12^). CCF_3_ signals do not found.
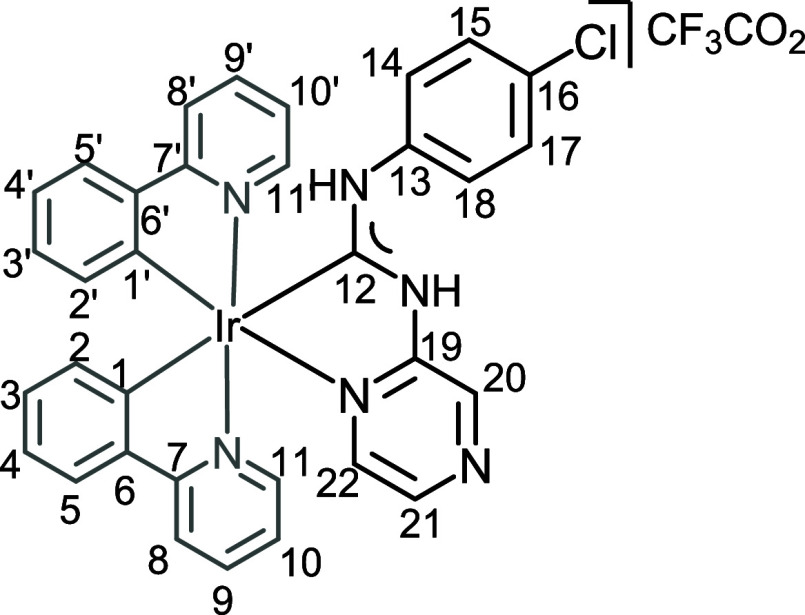


**4.** Yield 13 mg (74%), yellow solid. HRESI^+^-MS, *m*/*z*: calcd for C_34_H_25_F_3_IrN_6_ 767.1717, found 767.1749 [M + H]^+^. IR (KBr, cm^–1^): 3210 (low, wide), ν (N–H), 1656 (mid), ν­(C=N). ^1^H NMR (CDCl_3_, δ): 5.67 (d, *J*_H,H_ = 7.6 Hz, 1H, H_ppy_), 6.03 (d, *J*_H,H_ = 7.3 Hz, 1H, H_ppy_), 6.34 (t, *J*_H,H_ = 7.3 Hz, 1H, H_ADC_), 6.54 (t, *J*_H,H_ = 7.6 Hz, 1H, H_ppy_), 6.74–6.83 (m, 3H, H_ADC_), 6.88 (t, *J*_H,H_ = 7.3 Hz, 1H, H_ppy_), 6.97 (t, *J*_H,H_ = 7.6 Hz, 1H, H_ppy_), 7.03–7.10 (m, 2H, H_ppy_), 7.18–7.25 (m, 2H, H_ppy_), 7.30 (d, *J*_H,H_ = 3.3 Hz, 1H, H_ppy_), 7.55–7.59 (m, 2H, H_ppy_), 7.78–7.86 (m, 4H, H_ppy_), 8.08 (d, *J*_H,H_ = 3.3 Hz, 1H, H_ADC_), 8.71 (d, *J*_H,H_ = 6.0 Hz, 1H, H_ADC_), 9.00 (s, 1H, H_ADC_), 13.22 (s, 1H, N–H), 14.89 (s, 1H, N–H). ^13^C­{^1^H} NMR (CDCl_3_, δ): 119.62, 120.01, 120.56, 122.20, 122.98 (q, *J*_C,F_ = 4 Hz), 123.14, 123.89, 124.22, 124.43 (q, *J*_C,F_ = 4 Hz), 124.55, 129.32, 129.86, 130.25, 130.51, 130.71, 130.93, 135.27, 137.01, 137.98, 138.03, 139.32, 140.46, 141.31, 143.62, 148.49, 151.72, 152.99, 155.56, 163.18, 167.80, 168.83, 207.93 (C^12^). CCF_3_ signals do not found. ^19^F­{^1^H} NMR (CDCl_3_, δ): – 75.75, −62.60.
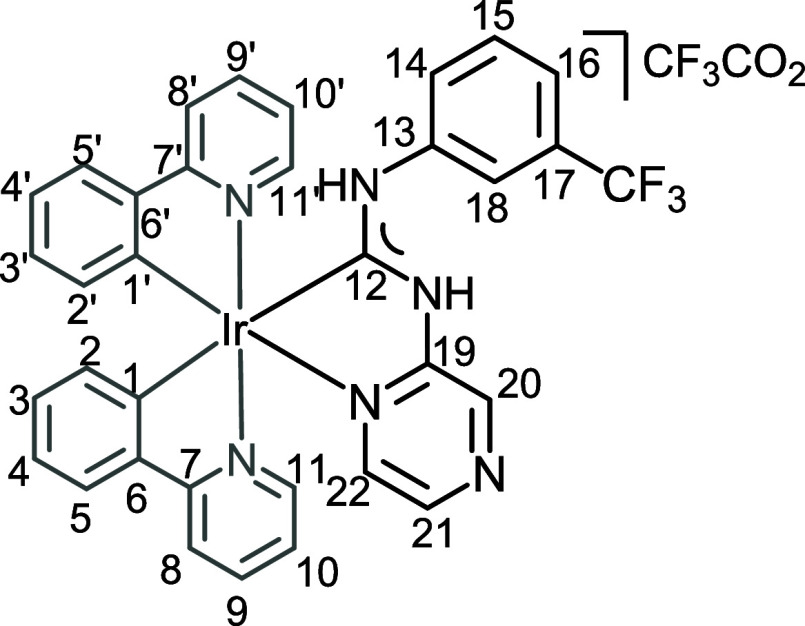


**5**. Yield 14 mg (84%), light-yellow solid. HRESI^+^-MS, *m*/*z*: calcd for C_34_H_26_ClIrN_5_ 732.1500, found 732.1509 [M + H]^+^. IR (KBr, cm^–1^): 3198 (low, wide), ν (N–H), 1666 (mid), ν­(C=N). ^1^H NMR (CDCl_3_, δ): 5.67 (d, *J*_H,H_ = 7.5 Hz, 1H, H_ppy_), 6.06 (d, *J*_H,H_ = 6.9 Hz, 1H, H_ppy_), 6.38 (t, *J*_H,H_ = 6.7 Hz, 1H, H_ppy_), 6.46 (d, *J*_H,H_ = 8.4 Hz, 2H, H_ADC_), 6.60 (d, *J*_H,H_ = 8.4 Hz, 2H, H_ADC_), 6.70–6.77 (m, 2H, H_Carb_), 6.87 (t, *J*_H,H_ = 7.2 Hz, 1H, H_ppy_), 6.93–7.01 (m, 2H, H_ppy_), 7.13 (t, *J*_H,H_ = 7.2 Hz, 1H, H_ppy_), 7.31 (d, *J*_H,H_ = 7.6 Hz, 1H, H_ppy_), 7.36 (d, *J*_H,H_ = 5.0 Hz, 1H, H_ADC_), 7.56–7.60 (m, 3H, H_ADC_), 7.67 (t, *J*_H,H_ = 7.0 Hz, 1H, H_ADC_), 7.74–7.85 (m, 4H, H_ppy_), 7.81 (d, *J*_H,H_ = 5.5 Hz, 1H, H_ppy_), 12.67 (s, 1H, N–H), 14.15 (s, 1H, N–H). ^13^C­{^1^H} NMR (CDCl_3_, δ): 111.48, 119.14, 119.26, 119.72, 119.97, 121.95, 122.73, 123.59, 124.14, 124.40, 127.25, 128.20, 129.93, 130.65, 130.74, 130.78, 132.78, 136.01, 136.72, 137.46, 139.72, 141.68, 143.61, 148.05, 148.68, 151.93, 152.98, 158.76, 164.47, 168.09, 168.87, 207.04 (C^12^). CCF_3_ signals do not found.
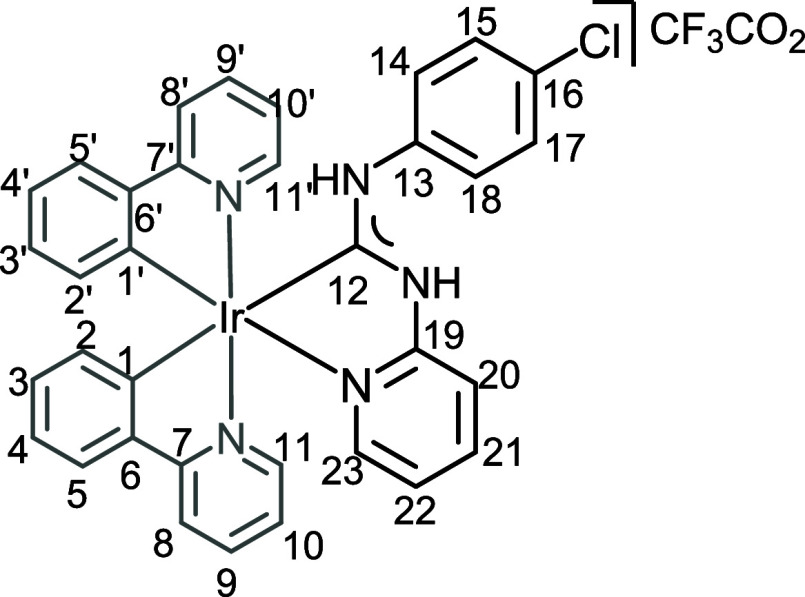


**6.** Yield 15 mg (85%), light-yellow solid. HRESI^+^-MS, *m*/*z*: calcd for C_35_H_26_F_3_IrN_5_ 766.1764, found 766.1789 [M + H]^+^. IR (KBr, cm^–1^): 3220 (low, wide), ν (N–H), 1656 (mid), ν­(C=N). ^1^H NMR (CDCl_3_, δ): 5.68 (d, *J*_H,H_ = 7.6 Hz, 1H, H_ppy_), 6.05 (d, *J*_H,H_ = 7.3 Hz, 1H, H_ppy_), 6.30 (t, *J*_H,H_ = 7.6 Hz, 1H, H_ppy_), 6.52 (t, *J*_H,H_ = 7.4 Hz, 1H, H_ppy_), 6.73–6.79 (m, 3H, H_ADC_), 6.84 (s, 1H, H_ADC_), 6.86 (t, *J*_H,H_ = 7.2 Hz, 1H, H_ppy_), 6.95 (t, *J*_H,H_ = 7.3 Hz, 1H, H_ppy_), 7.01 (td, *J*_H,H_ = 6.0 Hz, *J*_H,H_ = 2.6 Hz, 1H, H_ppy_), 7.06 (d, *J*_H,H_ = 7.5 Hz, 1H, H_ppy_), 7.17 (t, *J*_H,H_ = 6.2 Hz, 1H, H_ppy_), 7.23 (d, *J*_H,H_ = 7.8 Hz, 1H, H_ppy_), 7.37 (d, *J*_H,H_ = 5.2 Hz, 1H, H_ppy_), 7.57 (d, *J*_H,H_ = 7.7 Hz, 1H, H_ppy_), 7.60–7.63 (m, 2H, H_ADC_), 7.68 (t, *J*_H,H_ = 7.3 Hz, 1H, H_ADC_), 7.76–7.84 (m, 4H, H_ppy_), 8.77 (d, *J*_H,H_ = 5.6 Hz, 1H, H_ADC_), 12.81 (s, 1H, N–H), 14.25 (s, 1H, N–H). ^13^C­{^1^H} NMR (CDCl_3_, δ): 111.64, 118.98, 119.36, 119.73, 120.17, 121.93, 122.65, 123.21 (q, *J*_C,F_ = 4 Hz,), 123.53, 124.08, 124.18 (q, *J*_C,F_ = 3 Hz), 124.36, 129.20, 129.67, 130.49, 130.67, 130.73, 136.64, 137.49, 138.33, 139.60, 141.54, 143.64, 147.98, 148.62, 152.35, 153.02, 159.38, 164.88, 168.04, 168.95, 207.35 (C^12^). CCF_3_ signals do not found. ^19^F­{^1^H} NMR (CDCl_3_, δ): −75.69, −62.56.
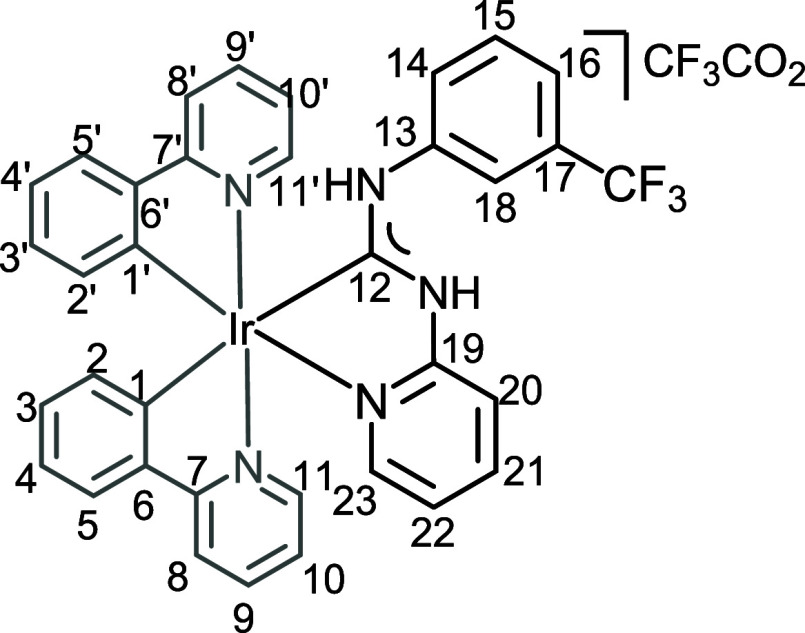


#### Synthesis of **3** with Deuterated Diaminocarbene Fragment

The solution of **3** (0.015 mmol) in CH_2_Cl_2_ was passed through a short column of potassium carbonate. Trifluoroacetic acid-d (CF_3_CO_2_D, 0.015 mmol) was added to the collected eluate. The solvent was then evaporated under reduced pressure, yielding the product as a yellow solid. The deuterated compound was used without further purification.

^1^H NMR (CDCl_3_, δ): 5.68 (d, *J*_H,H_ = 7.6 Hz, 1H, H^2^), 6.07 (d, *J*_H,H_ = 7.3 Hz, 1H, H^2′^), 6.40–6.51 (m, 3H), 6.63 (d, *J*_H,H_ = 8.5 Hz, 2H, H^15^), 6.77 (t, *J*_H,H_ = 7.2 Hz, 1H), 6.91 (t, *J*_H,H_ = 7.3 Hz, 1H), 6.97–7.07 (m, 2H), 7.17 (t, *J*_H,H_ = 7.3 Hz, 1H), 7.29–7.35 (m, 2H), 7.55 (d, *J*_H,H_ = 5.8 Hz, 1H), 7.60 (d, *J*_H,H_ = 7.8 Hz, 1H), 7.78–7.89 (m, 4H), 8.08 (d, *J*_H,H_ = 3.4 Hz, 1H), 8.66 (d, *J*_H,H_ = 5.6 Hz, 1H), 9.00 (s, 1H), 12.84 (s, 0.5H, N–H), 14.53 (s, 0.5H, N–H).
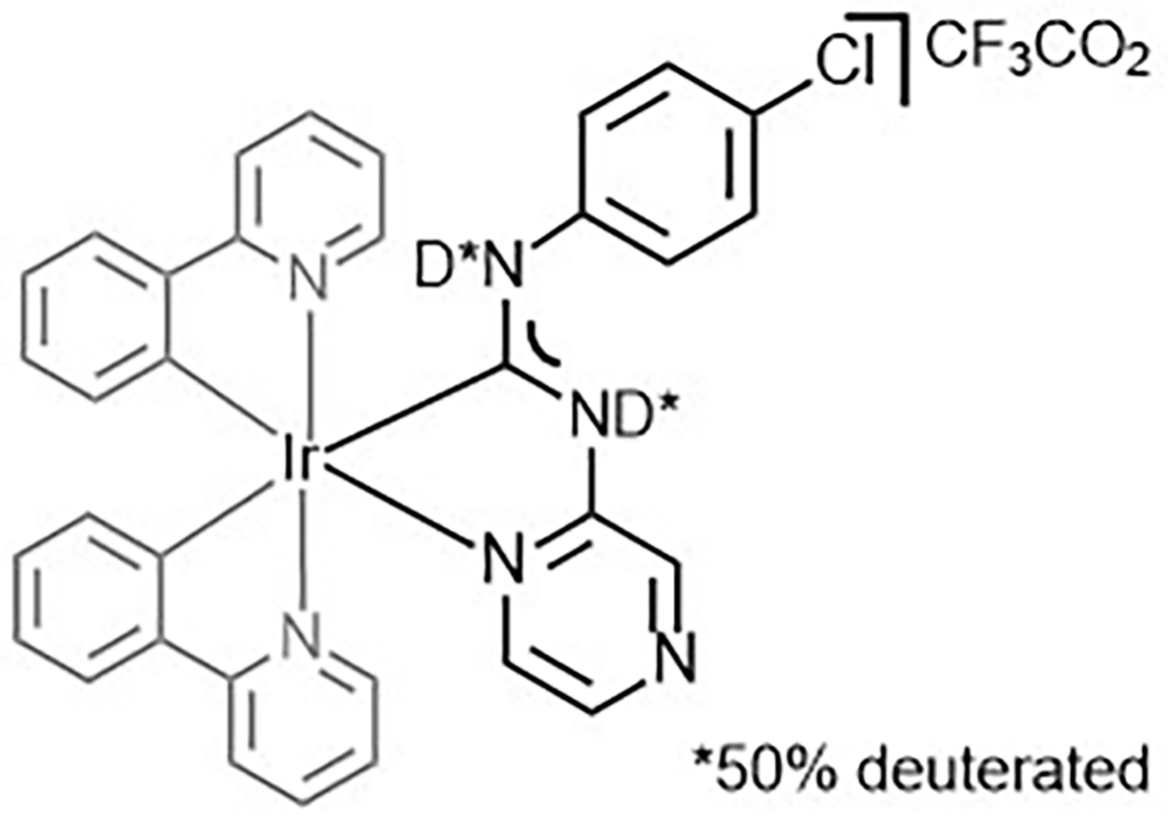


## Supplementary Material



## Notes

Experimental section and spectral, X-ray diffraction, experimental data and structural features discussion, theoretical calculations (PDF)
