# Supplementary material for: Photoinduced Tautomerisation of ESIPT-Capable Iridium(III) Complexes with Rationally Designed Acyclic Diaminocarbene Ligands
Source: Inorg Chem. 2026 Jan 13;65(3):1793–800. doi: 10.1021/acs.inorgchem.5c04206 (PMC12848967; doi:10.1021/acs.inorgchem.5c04206)
Supplement: Supplementary file 1 [file ic5c04206_si_001.pdf]

Supporting Information

Photoinduced Tautomerisation of ESIPT-capable Iridium(III) Complexes with Rationally Designed Acyclic Diaminocarbene Ligands

Polina O. Skripnyak,[a] Maria V. Kashina,[a] Anzhelika A. Eremina,[a] Sergei V. Tatarin,[b] Stanislav I. Bezzubov[b] Konstantin V. Luzyanin,*[c] and Mikhail A. Kinzhalov*[a]

[a]St Petersburg State University, 7–9 Universitetskaya Nab., 199034 Saint Petersburg, Russian Federation. E-mail: m.kinzhalov@spbu.ru

[b]Kurnakov Institute of General and Inorganic Chemistry, Russian Academy of Sciences, Leninskii Prosp. 31, 119991 Moscow, Russian Federation

[c]Department of Chemistry, University of Liverpool, Crown Street, Liverpool L69 7ZD, United Kingdom. E-mail: konstantin.luzyanin@liverpool.ac.uk

Table of content

S1. Materials and Instrumentation. 3
S2. Structure determination of new complexes **3**–**6**. 5
S3. X-ray diffraction studies. 7
S4. Photophysical data. 10
S5. Theoretical studies. 17
*S5.1. Geometry optimization of complexes **3**, $\mathbf{3^T}$ and **5**.* 17
S5.2. TD-DFT calculations. 23
S5.3. FMO analysis. 25
S6. NMR spectra for complexes **3**–**6**. 26
S7. FTIR spectra for complexes **3**–**6**. 32
S8. Mass spectra for complexes **3**–**6**. 34
S9. References. 36

S1. Materials and Instrumentation

Reagents and materials used. Solvents, $IrCl_3$•nH_2O (Aldrich), and organic reagents were obtained from commercial sources and used as received. $[Ir(ppy)_2(\mu\text{-}Cl)]_2$ was prepared by the known method,[1] that includes heating of $IrCl_3$•nH_2O at 110 °C with 2.5 equivs of 2-phenylpyridine in a 3:1 (v/v) mixture of 2-ethoxyethanol and deionized water. Isocyanides CNAr (Ar = C_6H_4-4-Cl, C_6H_4-3-CF) and complexes **1**, **2** were synthesized by the published procedure.[2-3]

Instrumentation and methods. Mass-spectra were acquired on Bruker micrOTOF spectrometer equipped with ESI source; MeOH was used as the solvent. The instrument was operated at positive ion mode using *m/z* range of 50–3000. The capillary voltage of the ion source was set at –4500 V (ESI^+) and the capillary exit at +(70–150) V. The nebulizer gas pressure was 0.4 bar and drying gas flow was 4.0 L/min. The most intensive peak in the isotopic pattern is reported. Infrared spectra were recorded on Shimadzu IRAffinity-1 FTIR instrument (4000–400 cm^{-1}, resolution 2 cm^{-1}) in KBr pellets. The UV/vis absorption spectra in CH_2Cl_2 solution were recorded on a Shimadzu UV-2500 spectrophotometer in a quartz cuvette with l = 1.0 mm. The luminescence and excitation spectra were recorded on a Fluorolog-3 (Horiba Jobin Yvon) instrument at 293 K. The solutions were degassed by blowing argon for 40 min. Lifetime measurements were achieved on Fluorat-02 Panorama spectrofluorimeter with a pulsed xenon lamp as an excitation source. The radiation decay curves were approximated be the $I(t) = I_1\left[1 - exp\left(-\frac{t}{\tau_1}\right)\right] + I_2\left[1 - exp\left(-\frac{t}{\tau_2}\right)\right] + \cdots$ equation with the OriginLab[4] software. The quantum yields were determined relative to a standard of $[Ru(bpy)_3](ClO_4)_2$ (Φ_{em} = 0.028).[5] The 1D (^1H, $^{13}C\{^1H\}$, $^{19}F\{^1H\}$), and 2D (^1H,^1H-COSY, ^1H,^{13}C-HMQC/HSQC and ^1H,^{13}C-HMBC, ^1H,^{15}N-HMQC/HSQC and ^1H,^{15}N-HMBC) NMR correlation experiments were acquired on a Bruker Avance III 400 spectrometer in $CDCl_3$ solvent at 25 C.

X-ray Structure Determinations. Single crystals of **4** were obtained by slow evaporation of its solution in CH_2Cl_2. A suitable crystal was mounted on a MiTeGen loop using crystallographic oil. Data were collected using a Rigaku XtaLAB Synergy, Single source at home/near, HyPix diffractometer equipped (monochromated MoKα radiation, λ = 0.71073 Å) at 299.49(10) K. The structure was solved with a ShelXT[6] structure solution program using Intrinsic Phasing and refined with a ShelXL[6] refinement package incorporated in the OLEX[2] program package[7] using Least Squares minimization. Empirical absorption correction was applied in CrysAlisPro[8] program complex using spherical harmonics, implemented in SCALE3 ABSPACK scaling algorithm. paper. CCDC number 2433604 contains the supplementary crystallographic data for

this paper. These data can be obtained free of charge from the Cambridge Crystallographic Data Centre via www.ccdc.cam.ac.uk/data_request/cif.

Computational details. The gas-phase calculations reported in this paper were performed within density functional theory (DFT),[9] using the hybrid functional B3LYP or the range-separated functional ωB97X.[10-11] The standard def2-TZVP basis set was applied for light elements and Stuttgart-Dresden effective core potential (ECP) for iridium atom[12] was used, as implemented in ORCA 4.2 suite of programs.[13] Triplet energies were calculated at the UB3LYP/def2-TZVP/SDD(Ir) theory level. For the TD-DFT analysis the 30 lowest-energy singlet excitations were calculated. Calculated absorption spectra were generated by processing the TD-DFT analysis results in ChemCraft[14] software.

S2. Structure determination of new complexes **3**–**6**

Complexes **3**–**6** were obtained as yellow solids. The compounds are stable in air and moisture and are soluble in common polar organic solvents. Complexes **3**–**6** were characterized by high resolution ESI$^+$-MS, IR, 1D (^{1}H, ^{13}C{^{1}H}, ^{19}F{^{1}H}) and 2D (^{1}H,^{1}H-COSY, ^{1}H,^{1}H-NOESY, ^{1}H,^{13}C-HMQC/^{1}H,^{13}C-HSQC, ^{1}H,^{13}C-HMBC, ^{1}H,^{15}N-HSQC, and ^{1}H,^{15}N-HMBC) NMR spectroscopy. The ESI$^+$-MS of **3**–**6** exhibit sets of peaks $[M + H]^+$ with the characteristic isotopic distribution.

In the IR spectra of complexes **3**–**6**, no strong bands are observed in the range of 2100–2500 cm^{-1}, indicating the conversion of the isocyanide ligand to a diaminocarbene. One weak significantly broadened band was observed around 3200 cm^{-1}, suggesting the presence of strong hydrogen bonding. In the ^{1}H NMR spectra of complexes **3**–**6**, the integration of signals confirms a 2:1 ratio of ppy to the diaminocarbene ligand, indicating the presence of two ppy ligands in **3**–**6**. Two NH protons resonate at 13–15 ppm, suggesting hydrogen bonding between these protons and the triflate anion. The addition of 2-aminoazaheterocycles to the isocyanide group results in a significant downfield shift of δ_C in ^{13}C{^{1}H} NMR spectra to the region specific for M–C$_{carbene}$ (δ_C 207.04–208.15 ppm) that confirms the formation of the ADC ligand.[15]

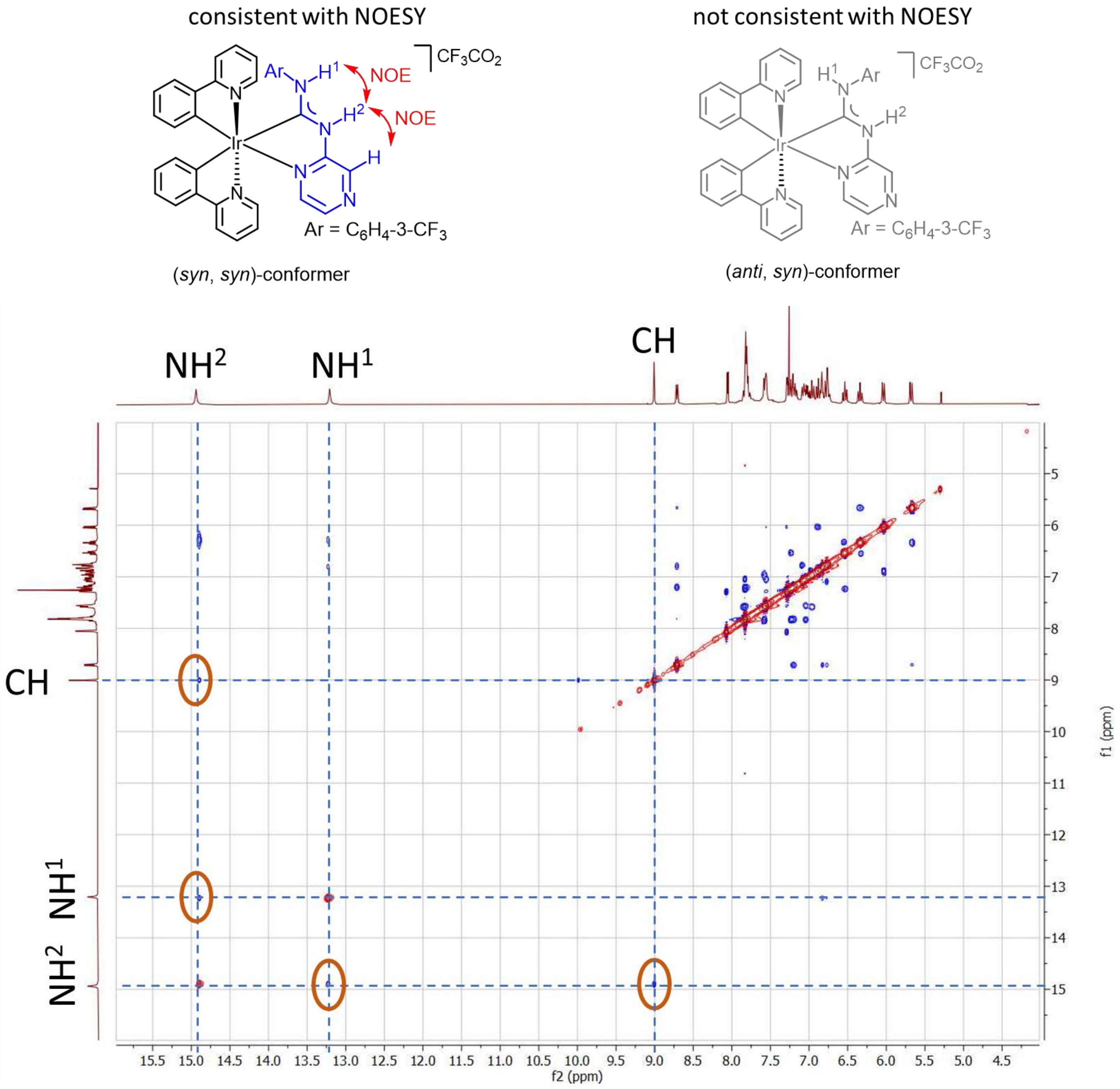

Figure S1. $^{1}H,^{1}H$-NOESY NMR spectrum of **4** in $CDCl_3$.

S3. X-ray diffraction studies

Table S1. Crystal data and structure refinement for **4**.

**Identification code**	**4**
CCDC	2433604
Empirical formula	$C_{36}H_{25}F_6IrN_6O_2$
Formula weight	879.82
Temperature/K	299.49(10)
Crystal system	Orthorhombic
Space group	Pbca
a /Å	17.7853(4)
b /Å	16.0458(2)
c /Å	22.4941(4)
α /°	90
β /°	90
γ /°	90
Volume /Å^3	6419.4(2)
Z	8
ρcalc g/cm^3	1.821
M /mm^{-1}	4.240
F(000)	3440.0
Crystal size /mm^3	0.3 × 0.2 × 0.1
Radiation	MoK$_\alpha$ (λ = 0.71073)
2Θ range for data collection /°	5.542 to 54.998
Index ranges	-23 ≤ h ≤ 23, -20 ≤ k ≤ 20, -29 ≤ l ≤ 29
Reflections collected	48814
Independent reflections	7357 [R_{int} = 0.0333, R_{sigma} = 0.0208]
Goodness–of–fit on F^2	1.208
Final R indexes [I≥2σ (I)]	R_1 = 0.0220, wR_2 = 0.0463
Final R indexes [all data]	R_1 = 0.0376, wR_2 = 0.0559
Largest diff. peak/hole /e·Å^{-3}	1.69/-0.73

Table S2. Selected bonds and angles in crystal structure of **4**.

	**Bond length, Å**		**Angle, °**
Ir1–N1	2.054(3)	N1–Ir1–N5	89.59(10)
Ir1–N2	2.053(3)	N1–Ir1–C23	100.26(11)
Ir1–N5	2.139(3)	N1–Ir1–C1	79.93(12)
Ir1–C23	2.092(3)	N2–Ir1–N1	172.56(10)
Ir1–C12	2.024(3)	N2–Ir1–N5	96.56(10)
Ir1–C1	2.060(3)	N2–Ir1–C23	85.21(11)
F3A–C2A	1.321(4)	N2–Ir1–C1	95.33(11)
O2A–C1A	1.241(4)	C23–Ir1–N5	77.26(11)
O1A–C1A	1.243(4)	C12–Ir1–N1	94.06(11)
F1A–C2A	1.328(4)	C12–Ir1–N2	79.75(12)
F2A–C2A	1.326(4)	C12–Ir1–N5	176.31(11)
C1A–C2A	1.558(5)	C12–Ir1–C23	102.53(12)
O1A–H4	1.919(2)	C12–Ir1–C1	85.23(12)
O2A–H3	1.953(2)	C1–Ir1–N5	94.94(11)
N4–H4	0.860(3)	C1–Ir1–C23	172.18(12)
N3–H3	0.860(3)	C7–N1–Ir1	116.0(2)
N4–C23	1.376(4)	C11–N1–Ir1	124.7(2)
N4–C30	1.372(4)	C18–N2–Ir1	116.1(2)
N3–C23	1.327(4)	C22–N2–Ir1	124.5(2)
N3–C24	1.425(4)	C30–N5–Ir1	114.2(2)
		C33–N5–Ir1	129.2(2)
		N4–C23–Ir1	112.4(2)
		N3–C23–Ir1	134.6(2)
		C17–C12–Ir1	114.6(2)
		C13–C12–Ir1	128.9(2)
		N3–C23–N4	112.1(3)
		O2A–C1A–O1A	129.7(3)
		N4–C30–C31	123.0(3)
		C23–N3–C24	125.5(3)

In crystal structure of **4**, Ir center adopts a slightly distorted octahedral coordination environment, and the two nitrogen atoms of the phenylpyridine ligands are in *trans*-position to each other. Ir–$C_{carbene}$ bond lengths are slightly increased (2.091–2.094 Å) relative to bonds between Ir and $C_{isocyanide}$ (1.991–2.019 Å[16-17]), which may indicate a slight decreased donor ability of the ADC ligand. The Ir–N_{ADC} bond lengths (2.111−2.152 Å) are greater than Ir–N_{ppy} (2.049–2.056 Å), which is related to the *trans* influence of the carbon atom in the cyclometallating ligand. The N–$C_{carbene}$–N fragment is nearly planar, and the angles around the carbon atom are close to 120° (111.3°−135.2°). The $C_{carbene}$–N bond lengths (1.327−1.378 Å) are

between those of a typical single CN bond (e.g., 1.469(10) Å in amines[18]) and a double CN bond (e.g., 1.279(8) Å in imines[18]). The diaminocarbene fragment adopts a *cis* conformation, where the aryl and heterocyclic substituents positioned on the same side of the N–$C_{carbene}$–N fragment. This conformation is stabilized by strong hydrogen bonds between $C_{carbene}$–NH groups and the counterion: the distance between the hydrogen and oxygen atoms (1.819−1.971 Å) are significantly less than the sum of the van der Waals radii (2.72 Å).

S4. Photophysical data

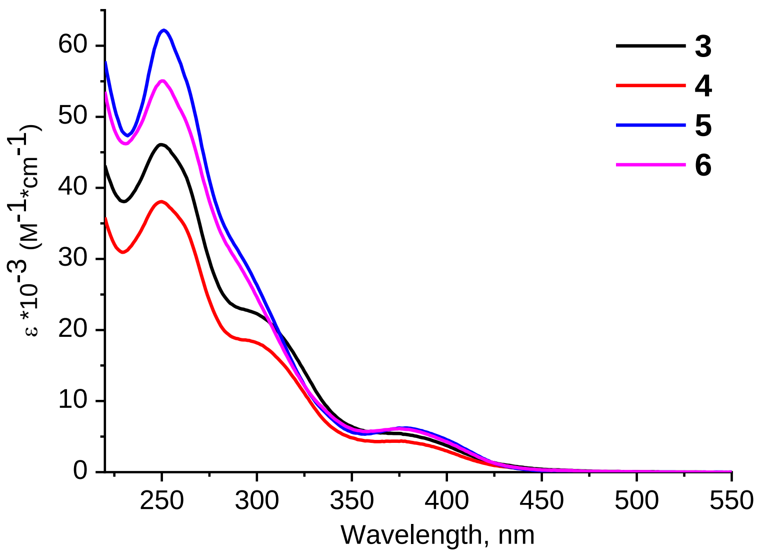

Figure S2. The UV-Vis absorption spectra for **3**–**6** in MeCN solution at RT.

Table S3. UV-Vis absorption data for **3**–**6**.

	Absorption: λ_{max}, nm		
**Complex**	in CH_2Cl_2 ($\varepsilon \times 10^{-3}$ $M^{-1}cm^{-1}$)	in MeCN ($\varepsilon \times 10^{-3}$ $M^{-1}cm^{-1}$)	solid state
**3**	302sh, 377 (7.83)	262sh, 290 (22.30), 377 (5.32)	258, 396sh, 580sh
**4**	292sh, 377 (7.83)	263sh, 290 (18.32), 379 (4.28)	
**5**	291sh, 381 (6.1)	263sh, 293sh, 378 (6.0)	270, 421sh, 592sh
**6**	291sh, 381 (5.6)	263sh, 292sh, 379 (6.1)	
**3 deuterated**	250, 285sh, 375 (6.58)		

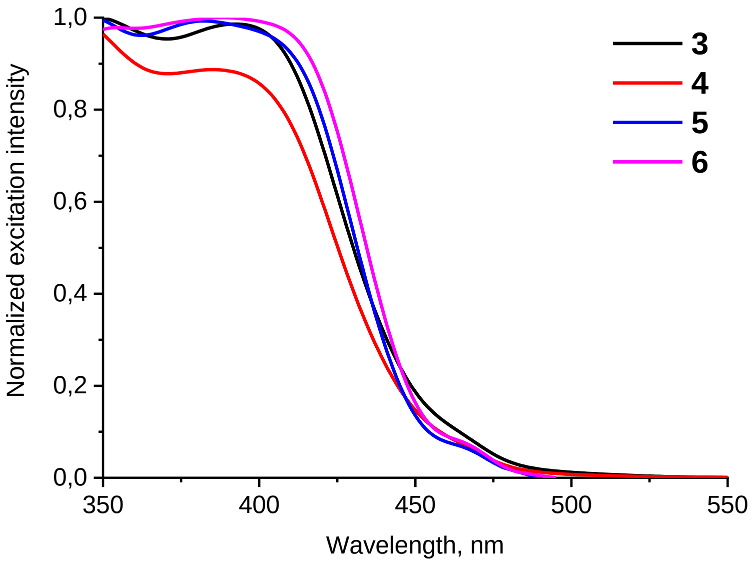

Figure S3. Excitation spectra of **3**–**6** in CH_2Cl_2.

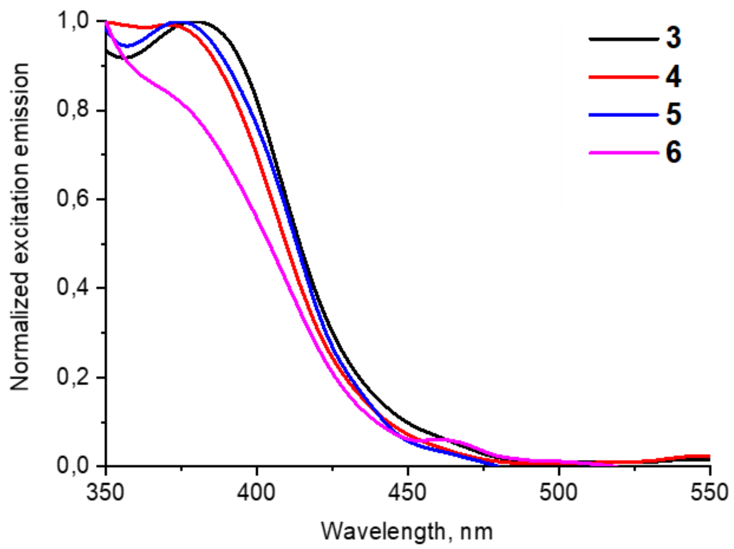

Figure S4. Excitation spectra of **3**–**6** in MeCN.

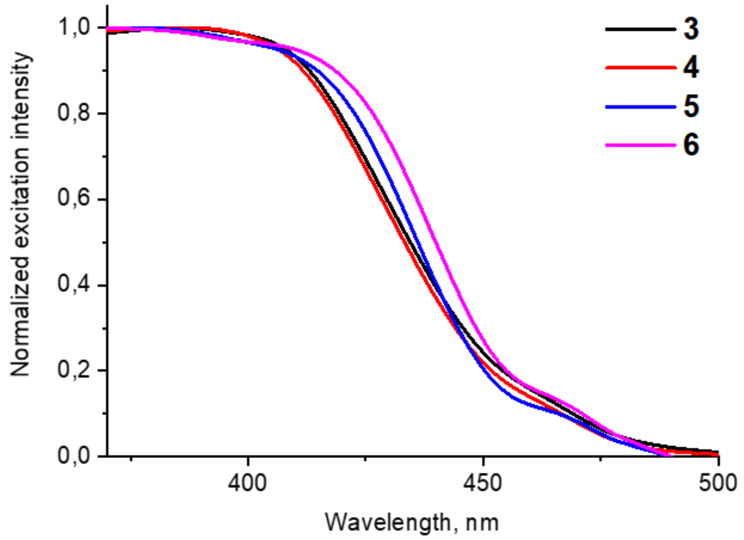

Figure S5. Excitation spectra of **3**–**6** in PMMA films.

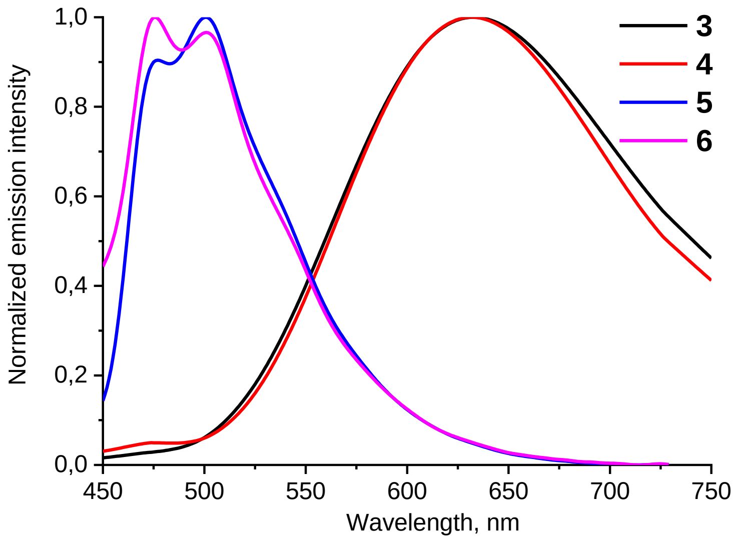

Figure S6. Emission spectra of **3**–**6** in MeCN.

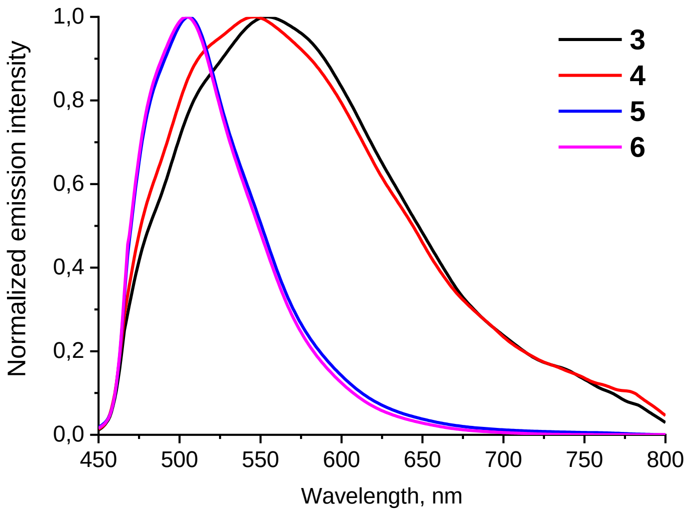

Figure S7. Emission spectra of **3**–**6** in PMMA films.

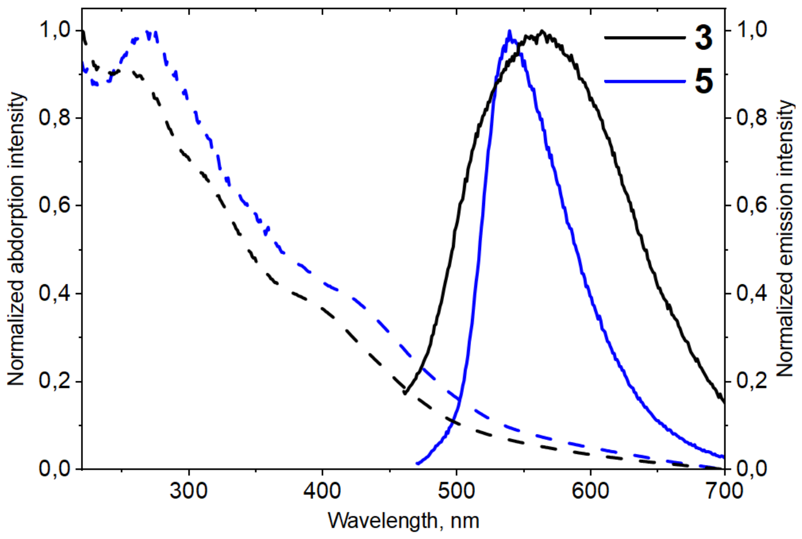

Figure S8. UV/vis-absorption (left) and emission (right) spectra of **3** and **5** in solid state.

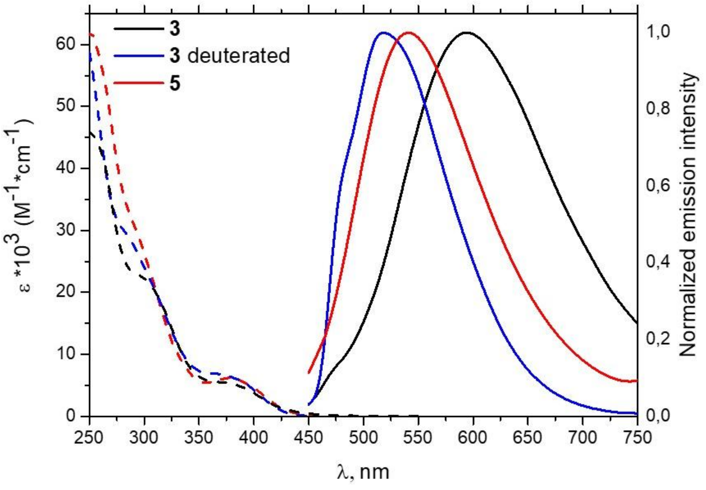

Figure S9. UV/vis-absorption (left) and emission (right) spectra of **3, 5** and deuterated analogue of **3** in CH_2Cl_2.

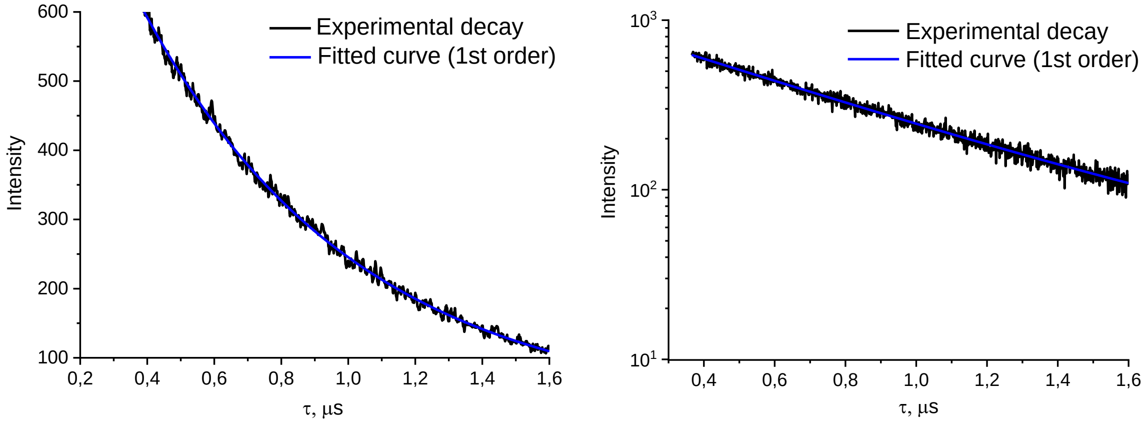

Figure S10. Photoluminescence decay plot of **3** in deaired conditions.

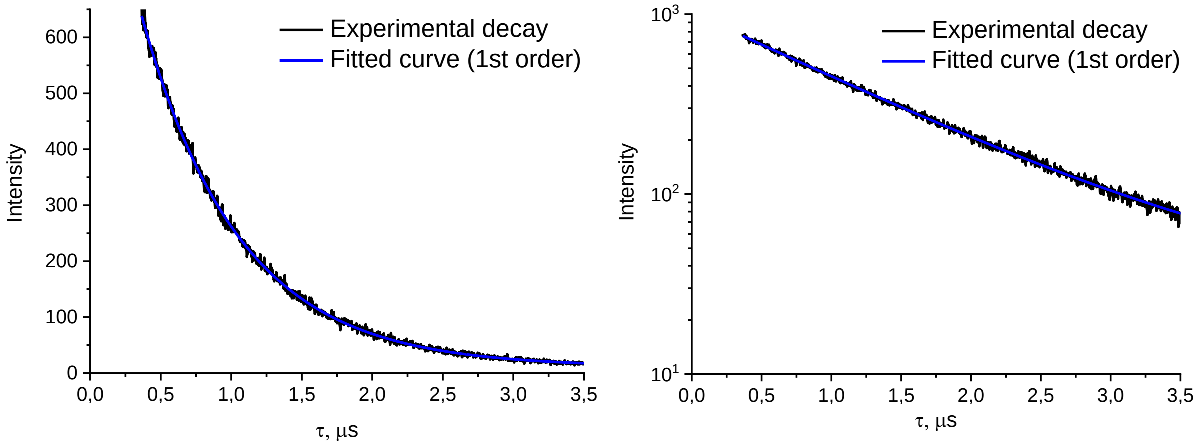

Figure S11. Photoluminescence decay plot of **4** in deaired conditions.

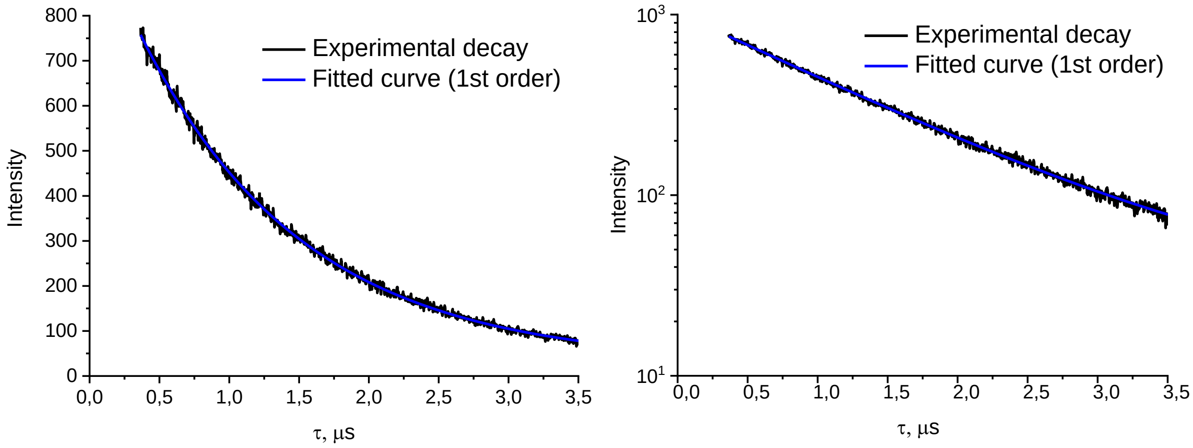

Figure S12. Photoluminescence decay plot of **5** in deaired conditions.

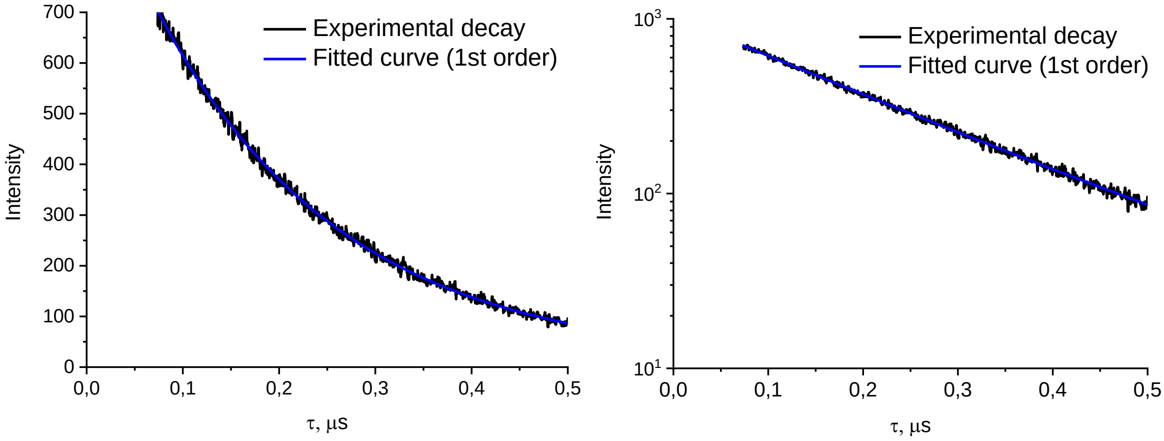

Figure S13. Photoluminescence decay plot of **6** in deaired conditions.

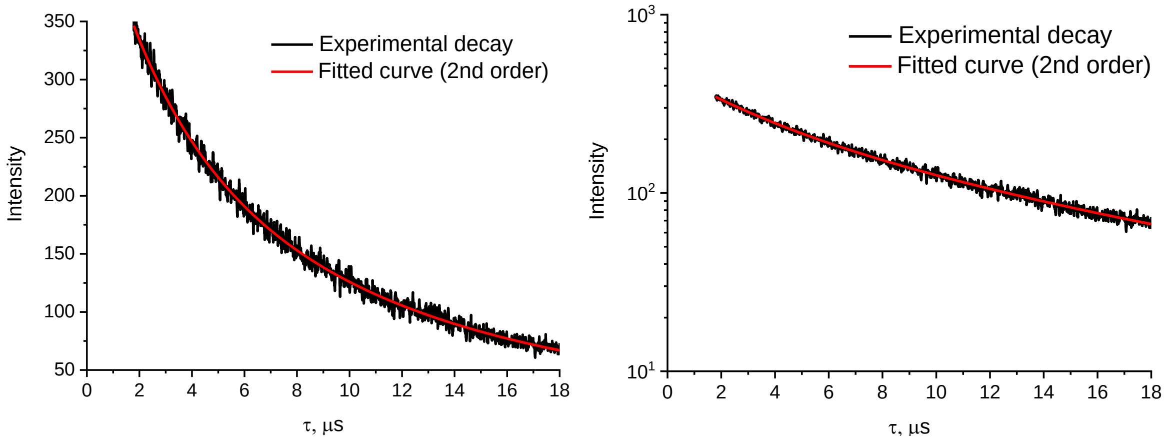

Figure S14. Photoluminescence decay plot of **3** in PMMA in deaired conditions.

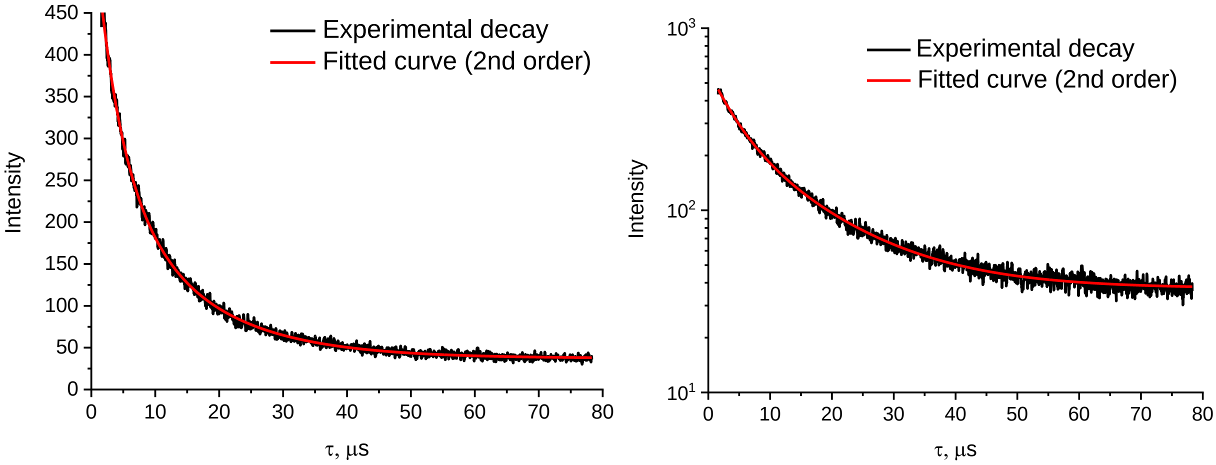

Figure S15. Photoluminescence decay plot of **4** in PMMA in deaired conditions.

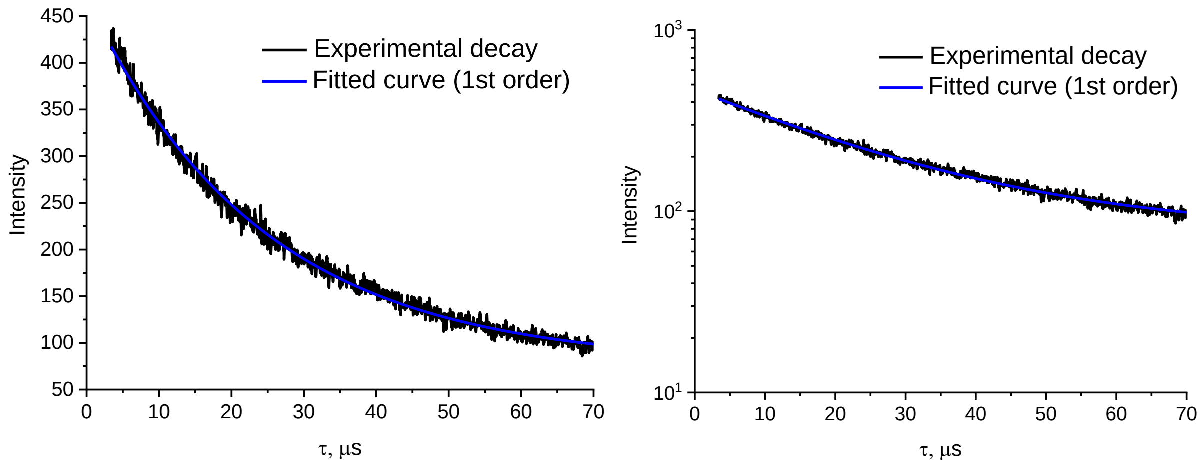

Figure S16. Photoluminescence decay plot of **5** in PMMA in deaired conditions.

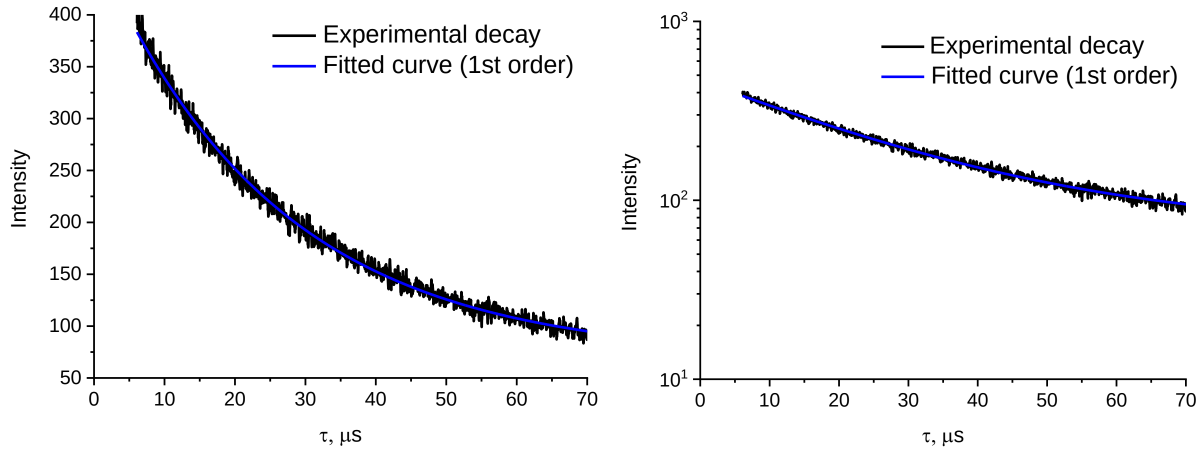

Figure S17. Photoluminescence decay plot of **6** in PMMA in deaired conditions.

Table S4. Phosphorescent excitation and emission data for **3**–**6** complexes at RT in different solvents.

Complex		λ_{ext}, nm	λ_{em}, nm	τ, μs	Φ_{em}	k_r, s^{-1}	k_{nr}, s^{-1}
**3**	CH_2Cl_2	350, 394	475sh, 594, 701sh	0.7	0.06	8.6×10^4	1.3×10^6
	PMMA	329, 388, 413sh	477sh, 510sh, 553, 587sh, 642sh	5.9 [2.9 (45), 10.8 (55)][a]	0.06	1.0×10^4	1.6×10^5
	MeCN	338sh, 382	633, 689sh		0.07		
	solid	450	560				
**3 deuterated**	CH_2Cl_2	322	481sh, 519, 544sh				
**4**	CH_2Cl_2	340, 390	480sh, 593, 703sh	0.7	0.06	8.6×10^4	1.3×10^6
	PMMA	312, 390	477sh, 513sh, 547, 589sh, 638sh	8.7 [4.0 (54), 13.5 (46)][a]	0.06	6.9×10^3	1.1×10^5
	MeCN	375	633, 692sh		0.10		
**5**	CH_2Cl_2	347, 386, 407sh	478, 505, 539sh	1.2	0.11	9.2×10^4	7.4×10^5
	PMMA	334, 389	479sh, 505, 538sh	24.1	0.21	8.7×10^3	3.3×10^4
	MeCN	246sh, 289, 373	476, 501, 534sh		0.10		
	solid	450	539				
**6**	CH_2Cl_2	350, 387, 412sh	479, 505, 540sh	1.9	0.17	8.9×10^4	4.4×10^5
	PMMA	330, 391, 413sh	477sh, 505, 540sh	25.6	0.26	1.0×10^4	2.9×10^4
	MeCN	253sh, 285, 317, 370	443sh, 476, 501, 534		0.14		

[a]Amplitude-weighted average lifetimes are given, as well as the different components in square brackets with relative amplitudes as percentages in parentheses.

S5. Theoretical studies

*S5.1. Geometry optimization of complexes **3**, **3^T** and **5**.*

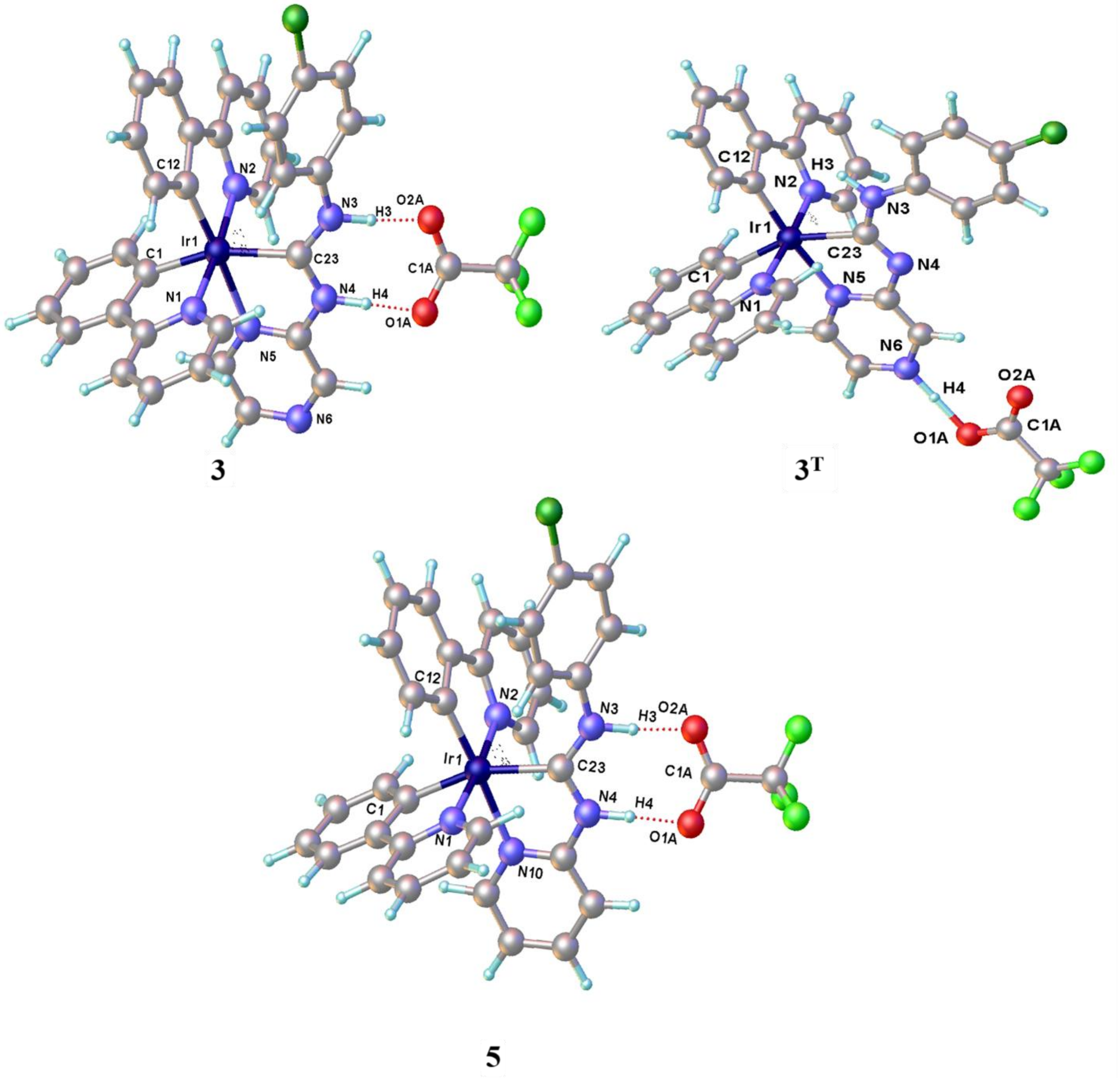

Figure S18. View of optimized structures of complexes **3**, **3^{T}** and **5** in the ground state with the selected atomic numbering schemes.

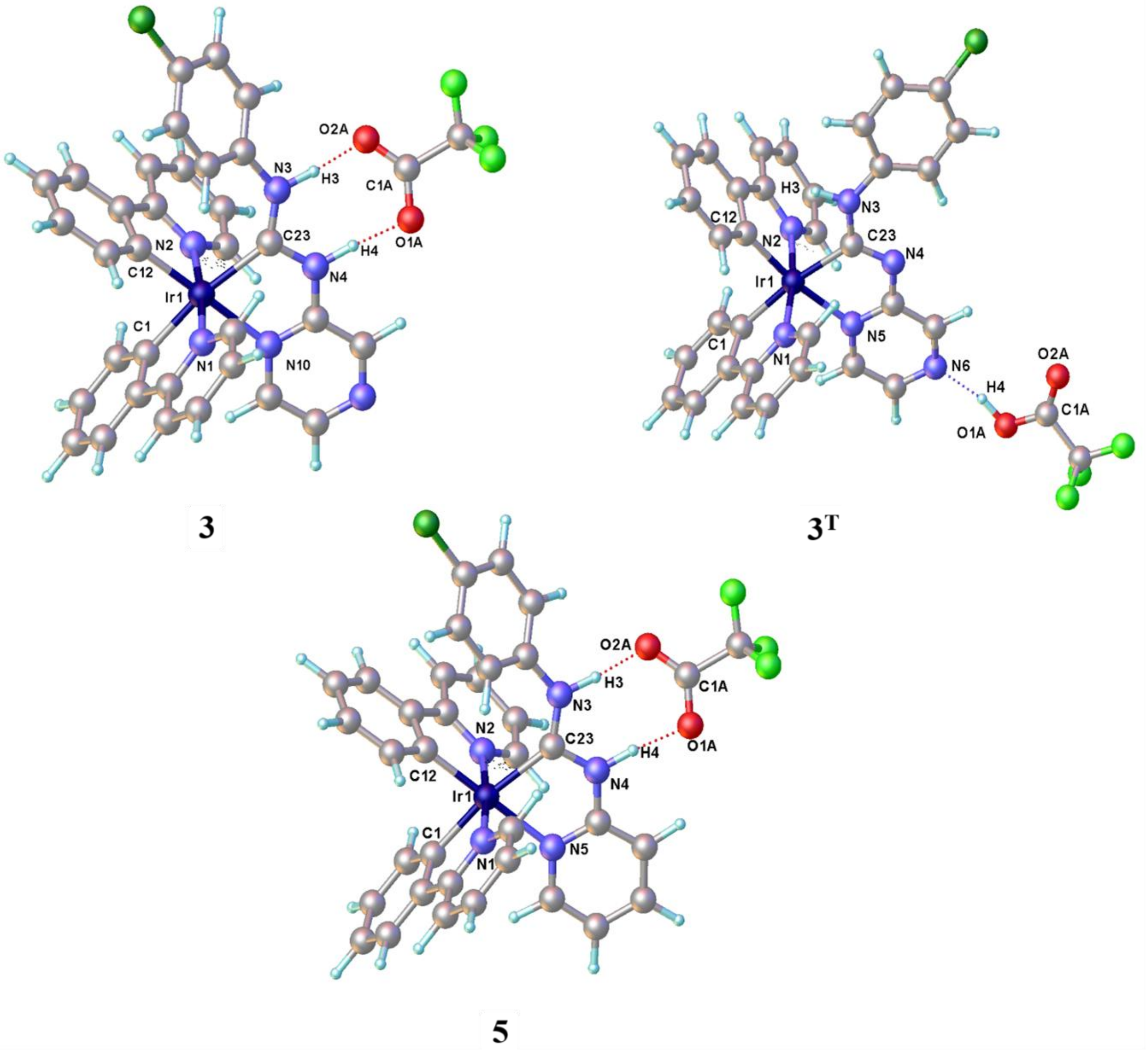

Figure S19. View of optimized structures of complexes **3**, $\mathbf{3^T}$ and **5** in the S1 state with the selected atomic numbering schemes.

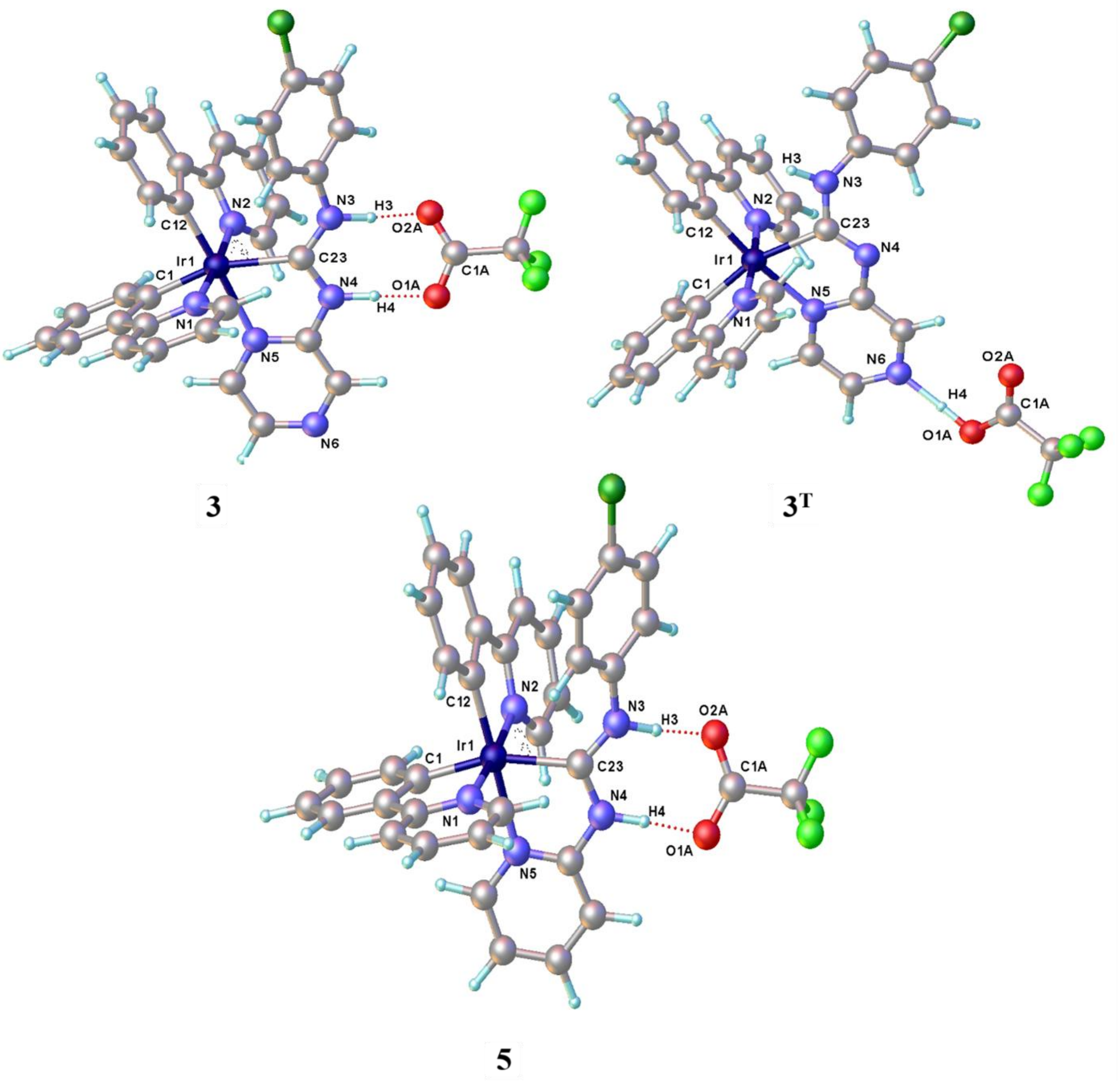

Figure S20. View of optimized structures of complexes **3**, $\mathbf{3^T}$ and **5** in the triplet state with the selected atomic numbering schemes.

Table S5. Selected bonds and angles in optimized geometries of different states for **3**, $\mathbf{3^T}$ and **5**.

Complex		S_0	S_1	T_1
**3**	**Bond length, Å**			
	Ir1–N1	2.089	2.070	2.066
	Ir1–N2	2.090	2.115	2.110
	Ir1–N5	2.219	2.286	2.314
	Ir1–C1	2.071	2.093	2.040
	Ir1–C12	2.033	1.964	1.995
	Ir1–C23	2.169	2.162	2.177
	O2A–C1A	1.249	1.249	1.250
	O1A–C1A	1.250	1.252	1.250
	O2A–H3	1.618	1.602	1.566
	O1A–H4	1.602	1.547	1.579
	N4–H4	1.073	1.083	1.078
	N4–C23	1.378	1.358	1.363
	N3–H3	1.065	1.076	1.076
	N3–C23	1.329	1.331	1.326
	**Angle, °**			
	N1–Ir1–N2	174.683	175.320	175.761
	N1–Ir1–N5	86.853	85.130	85.346
	N1–Ir1–C1	79.567	80.812	81.146
	N1–Ir1–C12	95.511	96.152	95.809
	N1–Ir1–C23	100.197	97.709	99.506
	N2–Ir1–N5	97.813	97.905	98.351
	N5–Ir1–C1	93.455	84.722	85.000
	C1–Ir1–C12	85.130	90.429	92.139
	C12–Ir1–C23	105.246	110.514	109.368
	N4–C23–N3	111.191	113.314	113.106
	O1A–H4–N4	175.120	172.669	177.119
	O2A–H3–N3	175.763	175.830	175.507
	O2A–C1A–O1A	128.148	128.257	128.126

Complex		S_0	S_1	T_1
3^T	**Bond length, Å**			
	Ir1–N1	2.090	2.093	2.088
	Ir1–N2	2.077	2.081	2.080
	Ir1–N5	2.094	2.247	2.205
	Ir1–C1	2.199	2.075	2.070
	Ir1–C12	2.028	2.006	2.028
	Ir1–C23	2.120	2.162	2.183
	O2A–C1A	1.205	1.230	1.213
	O1A–C1A	1.312	1.269	1.297
	O2A–H4	1.021	1.129	1.087
	H4–N6	1.654	1.416	1.476
	N3–C23	1.350	1.278	1.291
	N4–C23	1.360	1.373	1.376
	N3–H3	1.015	1.012	1.016
	**Angle, °**			
	N1–Ir1–N2	172.303	176.849	176.375
	N1–Ir1–N15	89.465	87.422	87.299
	N1–Ir1–C1	79.286	79.655	79.694
	N1–Ir1–C12	95.057	97.202	97.303
	N1–Ir1–C23	98.284	92.751	94.184
	N2–Ir1–C12	79.972	80.626	80.149
	N5–Ir1–C1	97.305	86.151	90.884
	C1–Ir1–C12	91.346	97.917	94.776
	N3–C23–N4	118.788	123.978	125.063
	O1A–H4–N6	167.366	178.458	177.458
	O1A–C1A–O2A	128.127	129.448	128.241

**5**	**Bond length, Å**			
	Ir1–N1	2.087	2.071	2.063
	Ir1–N2	2.090	2.113	2.115
	Ir1–N5	2.226	2.292	2.307
	Ir1–C1	2.072	2.094	2.039
	Ir1–C12	2.034	1.965	2.000
	Ir1–C23	2.166	2.158	2.170
	O70–C69	1.248	1.248	1.250
	O71–C69	1.251	1.252	1.250
	O70–H13	1.664	1.616	1.579
	O71–H68	1.628	1.570	1.578
	N11–H68	1.066	1.077	1.072
	N12–H13	1.063	1.073	1.072
	**Angle, °**			
	N1–Ir1–N2	174.855	175.390	175.708
	N1–Ir1–N5	87.519	85.351	86.471
	N1–Ir1–C1	79.542	80.766	81.214
	N1–Ir1–C12	95.628	96.260	95.694
	N1–Ir1–C23	100.141	97.657	99.755
	N2–Ir1–C12	79.768	80.520	80.279
	N2–Ir1–C1	97.672	95.884	97.375
	N2–Ir1–C23	83.364	86.594	82.995
	N5–Ir1–C1	93.769	85.160	85.972
	N5–Ir1–C12	176.393	174.706	176.884
	C1–Ir1–C12	85.091	90.114	92.136
	N4–C23–N3	111.167	113.305	112.943
	O1A–H4–N4	174.034	172.179	176.459
	O2A–H3–N3	175.893	175.938	175.488
	O1A–C1A–O2A	128.269	128.368	128.229

S5.2. TD-DFT calculations

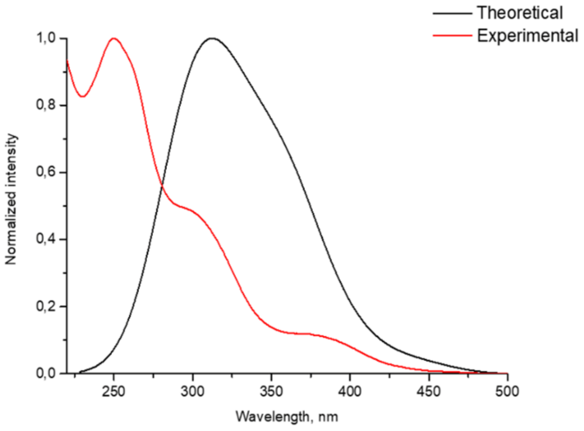

3

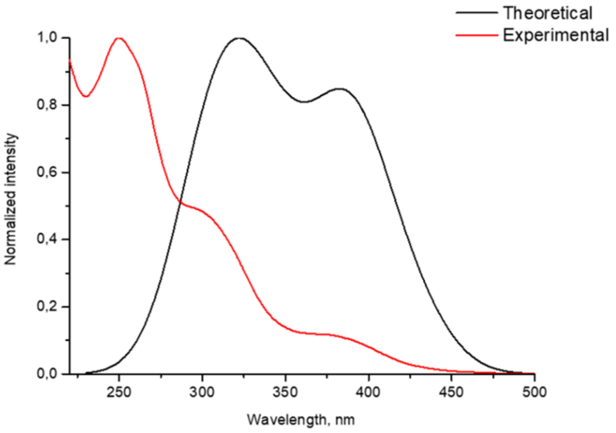

3^{T}

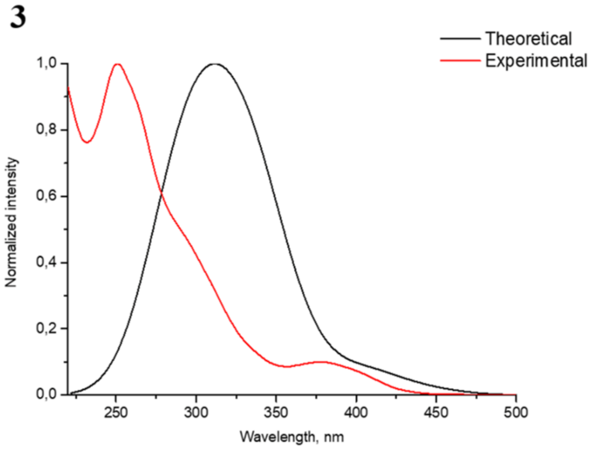

5

Figure S21. Experimental and the TD-DFT calculated absorption spectra for **3**, **3^{T}** and **5** in CH_3CN. Absorption spectra were calculated from fully optimized geometry in B3LYP, using def2-TZVP for light atoms and ECP for Ir atom and are plotted by broadening of vertical electronic transitions by Gauss.

Table S6. TD-DFT transition energies to the low-lying singlet states with significant oscillator strengths *f*, the main configurations and the characters of the complexes **3**, $\mathbf{3^T}$ and **5**. Absorption spectra were calculated from fully optimized geometry in B3LYP, using def2-TZVP for light atoms and ECP for Ir atom.

	**3**				$\mathbf{3^T}$			
**State**	**Energy, eV**	***λ*, nm**	***f***	**Transitions**	**Energy, eV**	***λ*, nm**	***f***	**Transitions**
**S1**	2.80	443	0.0032	H → L (88%)	2.70	459	0.0007	H → L (97%)
**S2**	2.80	442	0.0092	H → L+1 (88%)	2.91	426	0.0434	H → L+1 (86%)
**S3**	3.09	401	0.0311	H → L+2 (93%)	2.98	416	0.0084	H–2 → L (55%)<br>H → L+2 (30%)
**S4**	3.30	375	0.0058	H → L+3 (93%)	2.99	415	0.0093	H–2 → L (28%)<br>H → L+2 (55%)
**S5**	3.37	368	0.0107	H–1 → L (30%)<br>H → L+1 (57%)	3.18	389	0.1851	H–1 → L (79%)
**S6**	3.39	366	0.1053	H–1 → L (57%)<br>H → L+1 (26%)	3.26	380	0.0060	H-1 → L+1 (70%)<br>H-1 → L+2 (23%)
**S7**	3.52	352	0.0176	H–3 → L (38%)<br>H-1 → L+2 (24%)	3.32	373	0.0026	H–1 → L+2 (35%)<br>H → L+3 (22%)
**S8**	3.53	351	0.0094	H–1 → L+2 (34%)<br>H → L+4 (46%)	3.34	371	0.0095	H → L+3 (62%)

	**5**			
**State**	**Energy, eV**	***λ*, nm**	***f***	**Transitions**
**S1**	2.78	445	0.0079	H → L (96%)
**S2**	3.04	408	0.0277	H→ L+1 (89%)
**S3**	3.13	396	0.0078	H → L+2 (91%)
**S4**	3.29	377	0.0068	H → L+3 (90%)
**S5**	3.35	371	0.0053	H–1 → L (85%)
**S6**	3.51	354	0.0104	H-1→ L+1 (81%)
**S7**	3.52	352	0.0061	H→ L+4 (87%)
**S8**	3.55	349	0.0521	H-3→ L (34%)<br>H-2→ L (44%)

Table S7. TD-DFT transition energies to the T1→S0 transition of the complexes **3**, $\mathbf{3^T}$ and **5**, calculated from fully optimized geometry in B3LYP, using def2-TZVP for light atoms and ECP for Ir atom.

**Complex**	**Energy, eV**	***λ*, nm**
**3**	2.65	468
$\mathbf{3^T}$	2.47	502
**5**	2.64	469

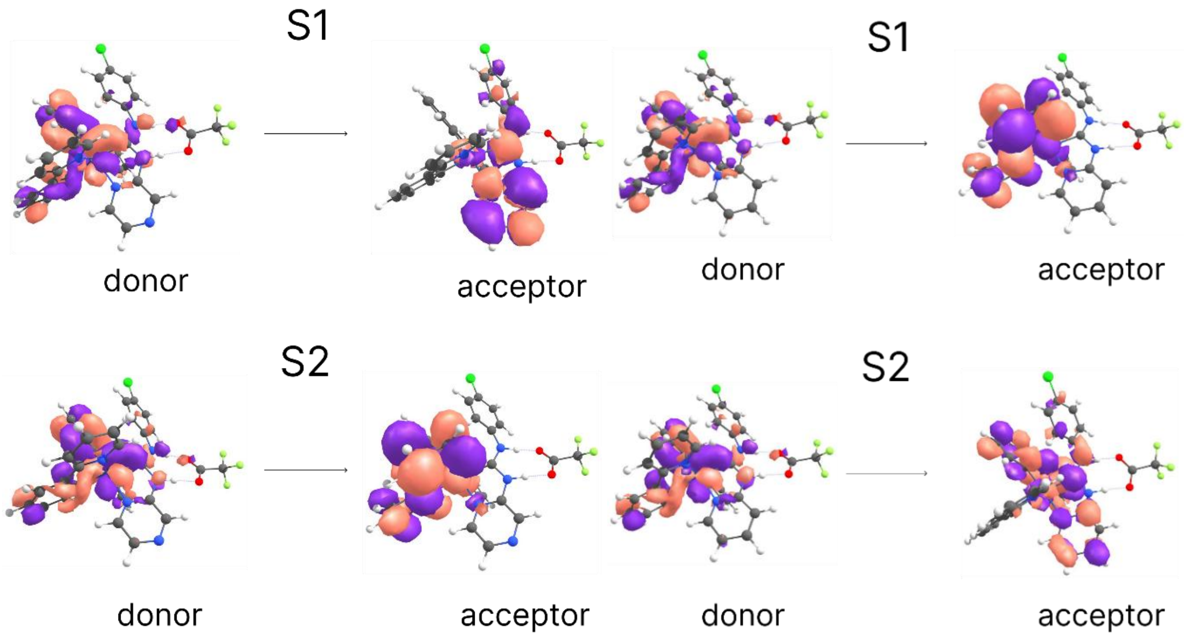

Figure S22. Natural transition orbitals (NTOs) representing S1 and S2 lowest energy transitions for complexes **3** (left) and **5** (right).

S5.3. FMO analysis

Table S8. Spin density contributions of certain structural fragments in the lowest triplet excited state for **3**, $\mathbf{3^T}$ and **5**.

**Complex**	**M, %**	**C^N, %**	**Carbene moieties, %**
**3**	23.1	70.2	6.7
$\mathbf{3^T}$	7.6	11.7	80.7
**5**	22.3	64.3	13.4

Table S9. Relative energies of S0, S1 and T1 states for **3** and $\mathbf{3^T}$ calculated using B3LYP and ωB97X functionals

**Complex**	**S0, eV**	**S1, eV**	**T1, eV**
**3** (B3LYP)	–5.8	–2.3	–3.2
$\mathbf{3^T}$ (B3LYP)	–5.6	–2.2	–3.4
**3** (ωB97X)	–8.1	–0.3	–5.1
$\mathbf{3^T}$ (ωB97X)	–7.9	–0.2	–5.6

S6. NMR spectra for complexes **3**–**6**

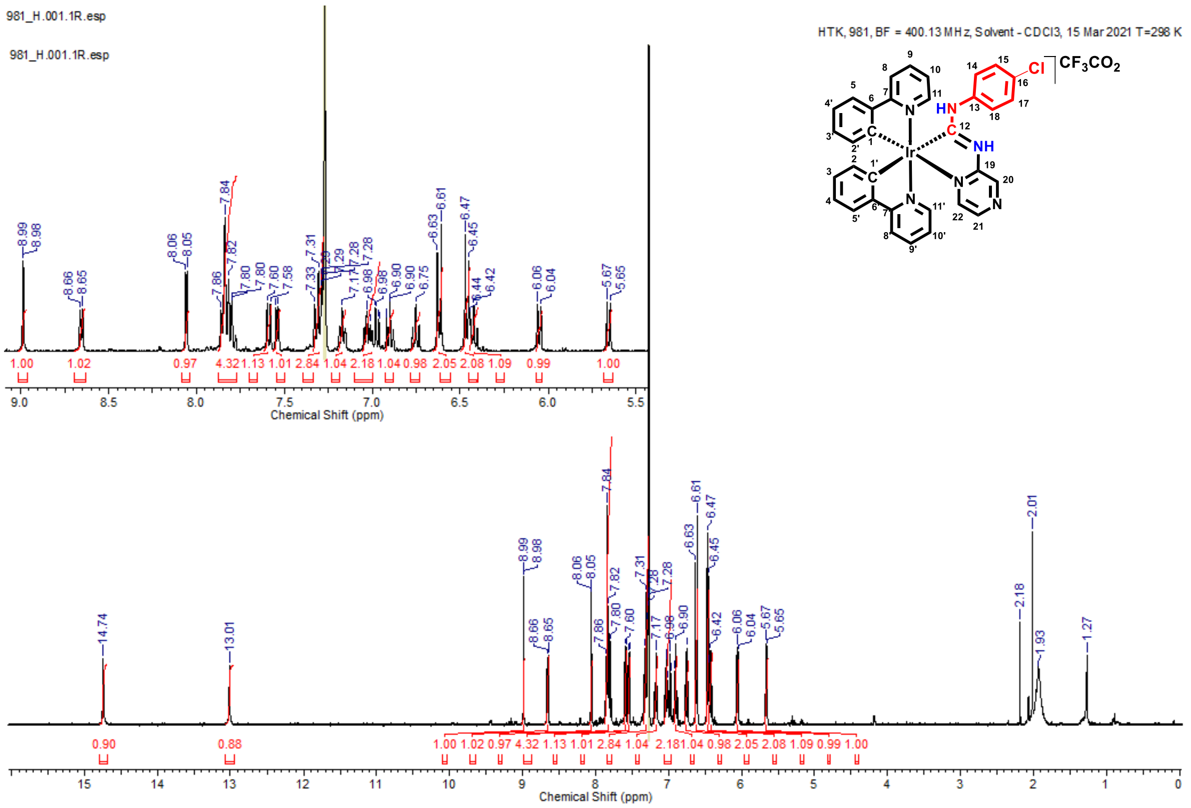

Figure S24. ^{1}H NMR spectra for **3** in $CDCl_3$.

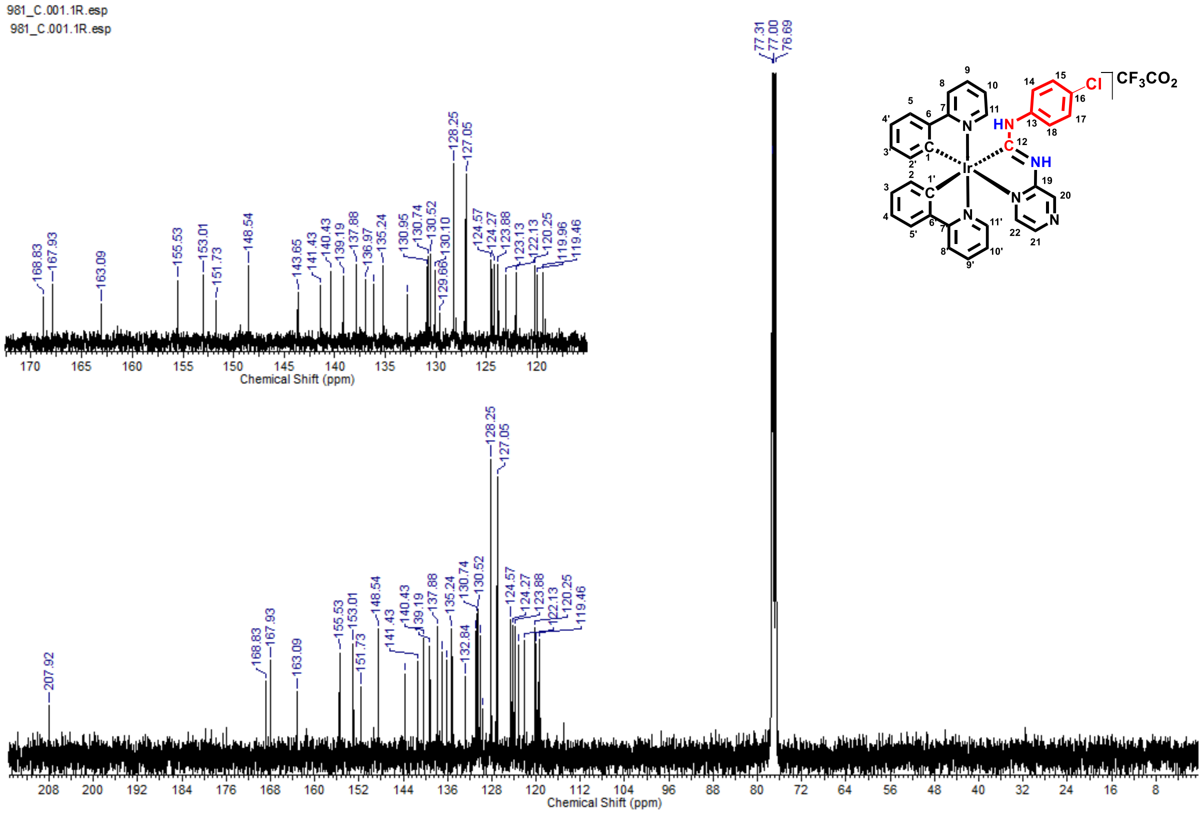

Figure S25. $^{13}C\ \{^{1}H\}$ NMR spectra for **3** in $CDCl_3$.

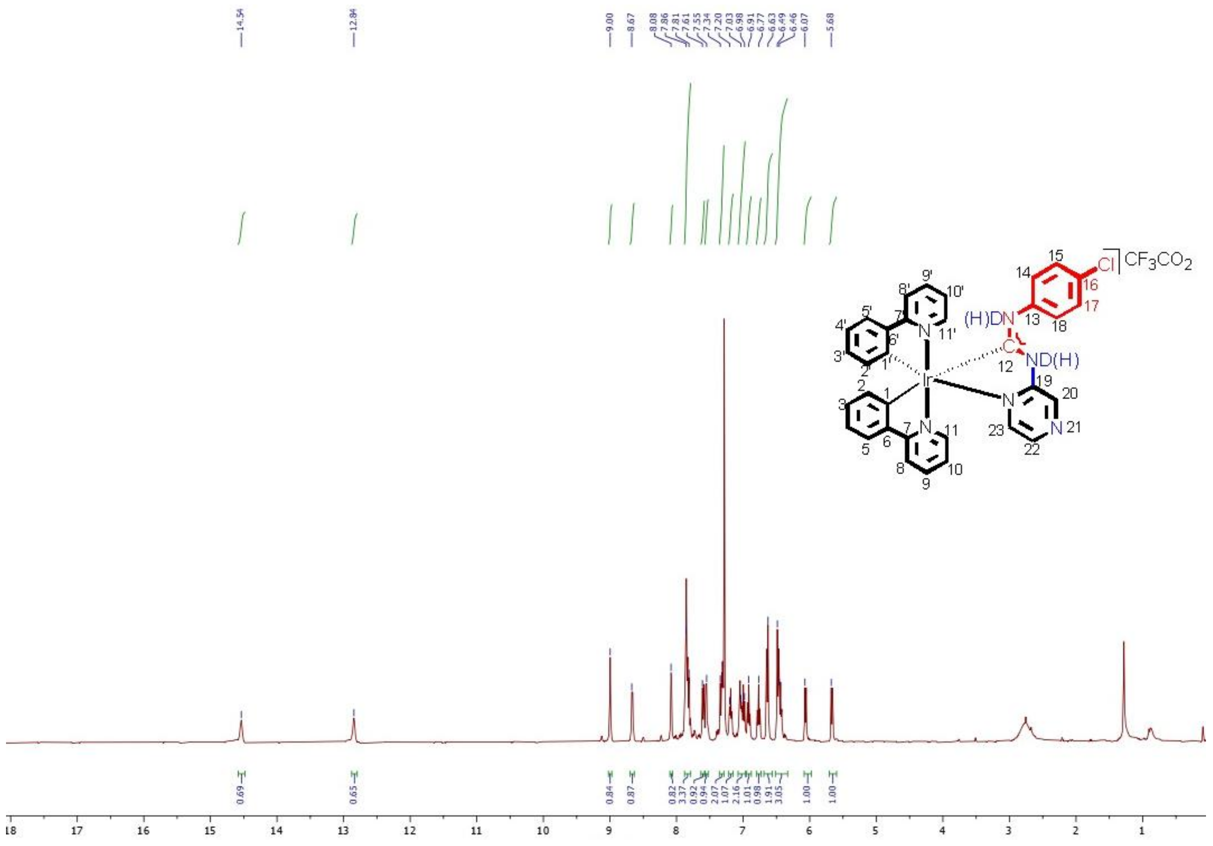

Figure S26. ^{1}H NMR spectra for deuterated **3** in $CDCl_3$.

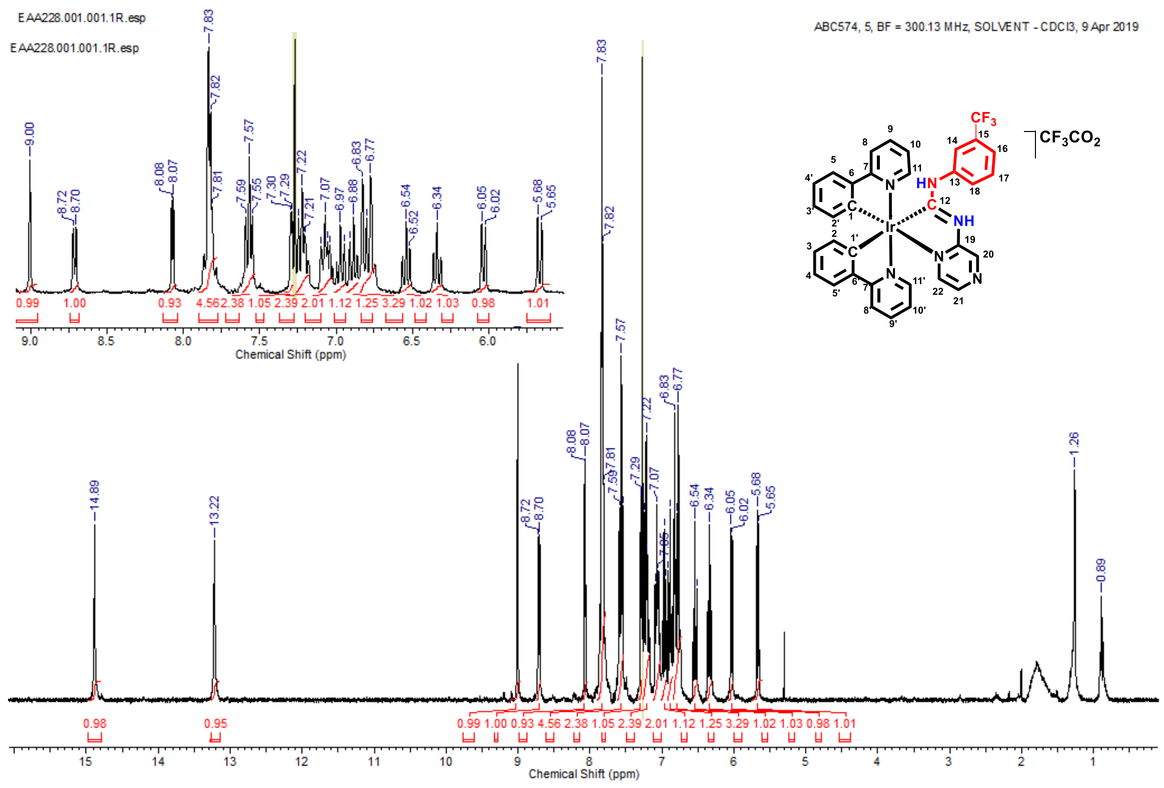

Figure S27. ^{1}H NMR spectra for **4** in $CDCl_3$.

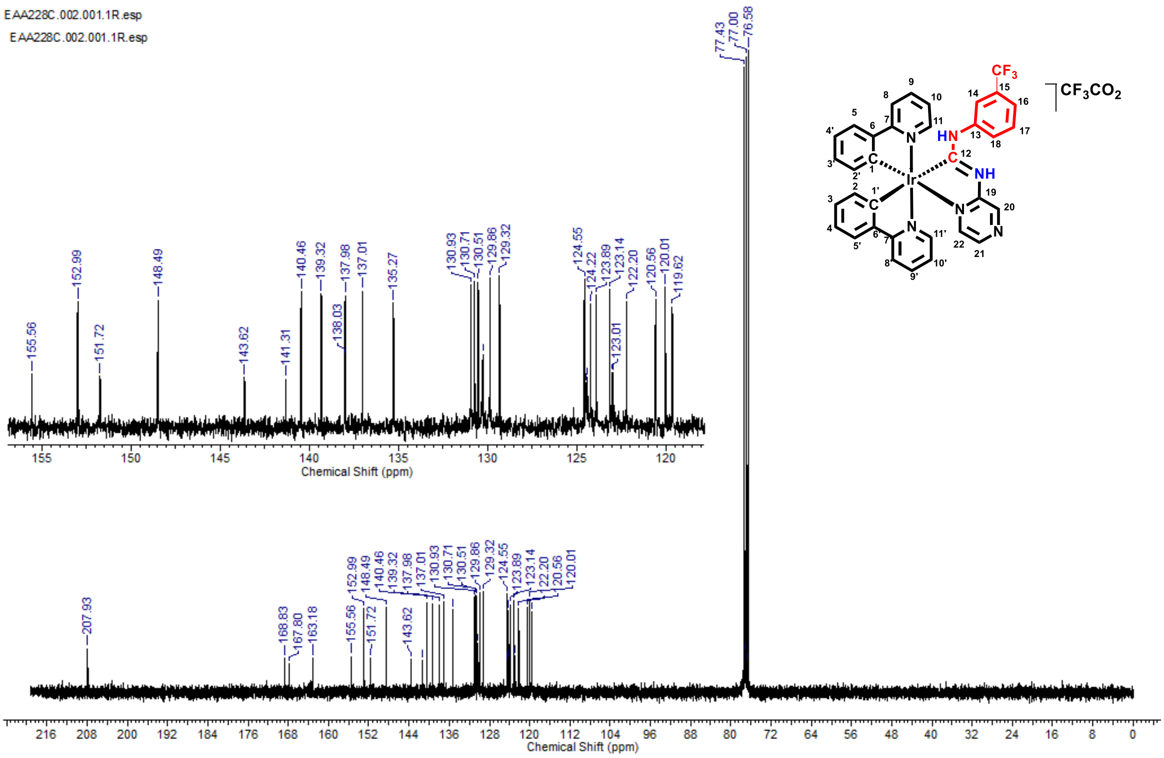

Figure S28. ^{13}C {^{1}H} NMR spectra for **4** in $CDCl_3$.

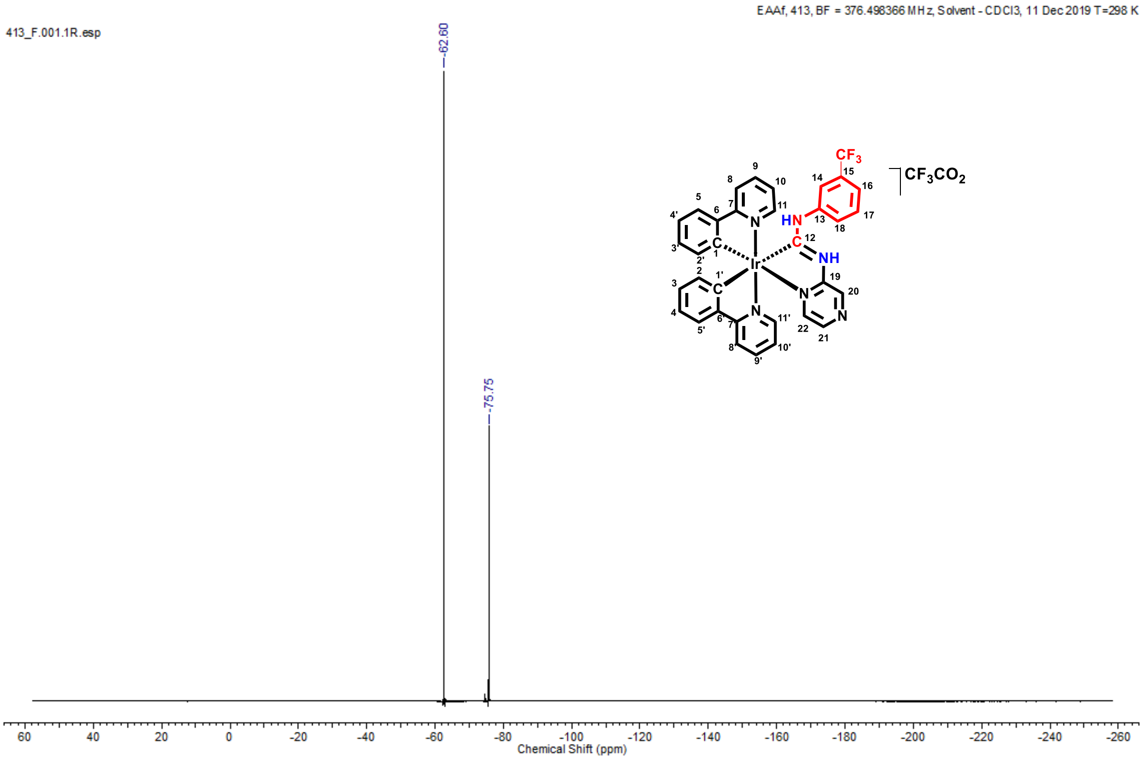

Figure S29. ^{19}F {^{1}H} NMR spectra for **4** in $CDCl_3$.

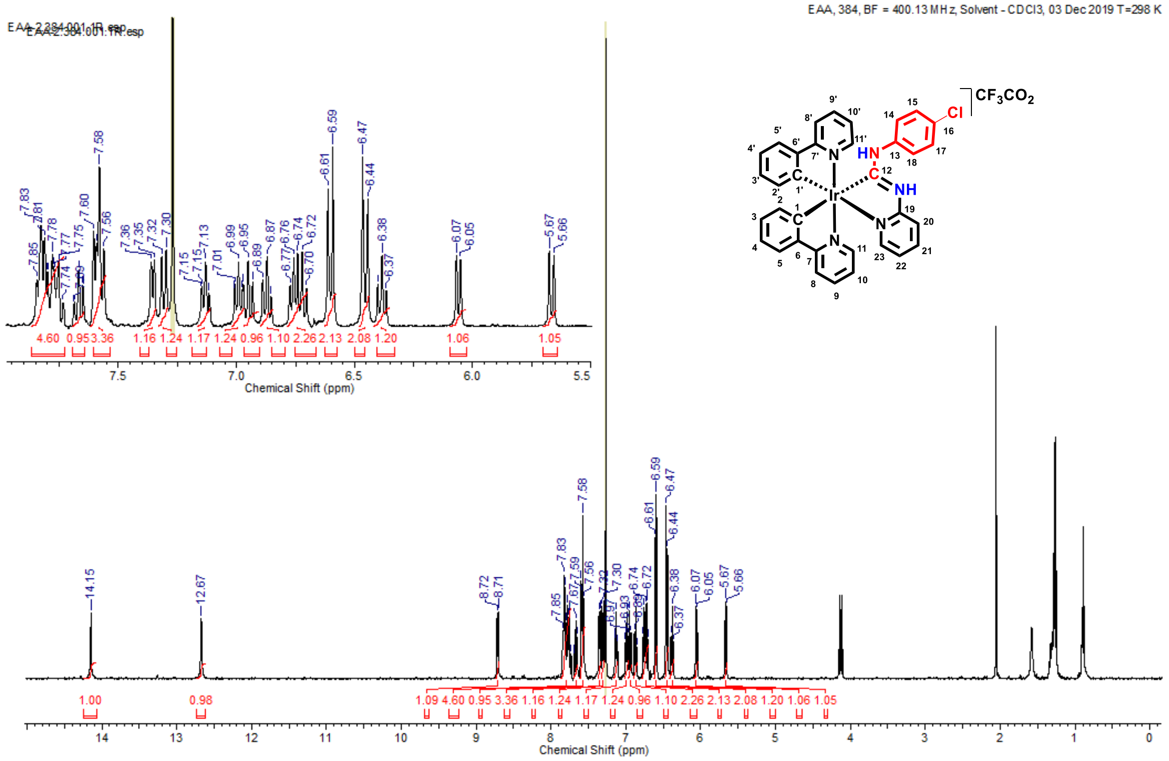

Figure S30. ^{1}H NMR spectra for **5** in $CDCl_3$.

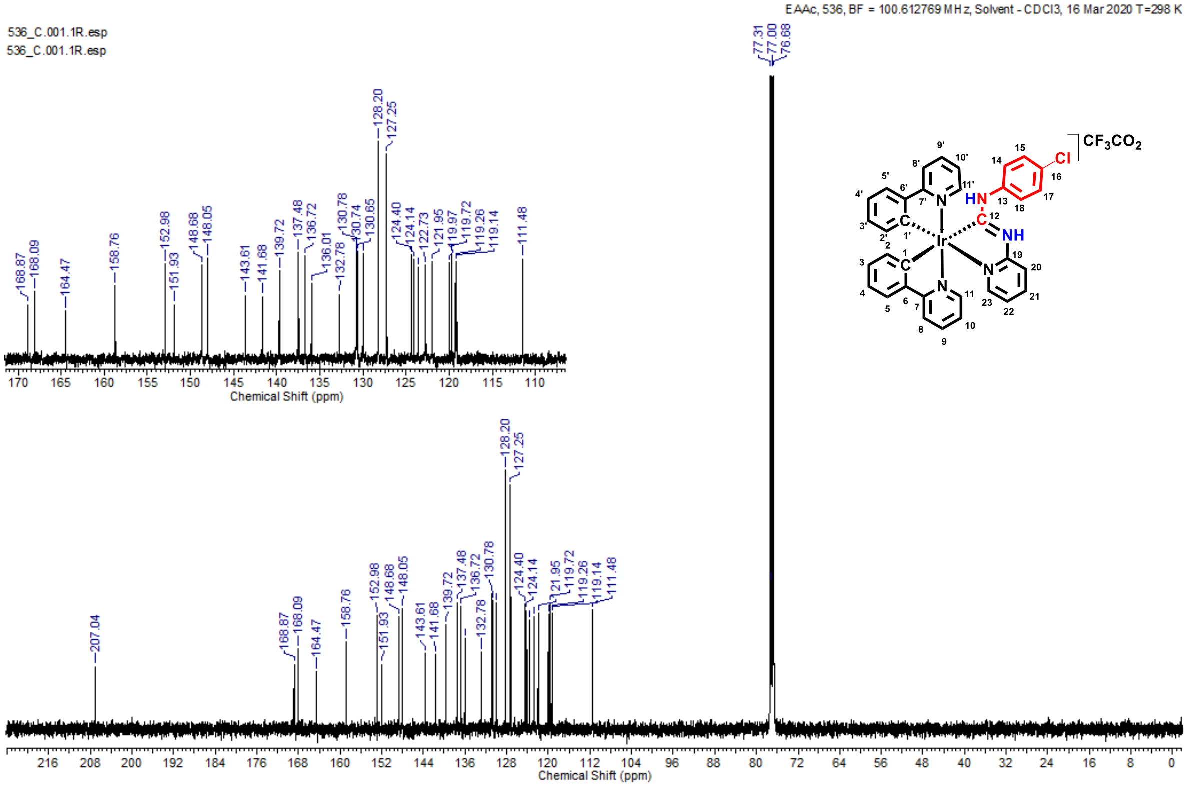

Figure S31. ^{13}C {^{1}H} NMR spectra for **5** in $CDCl_3$.

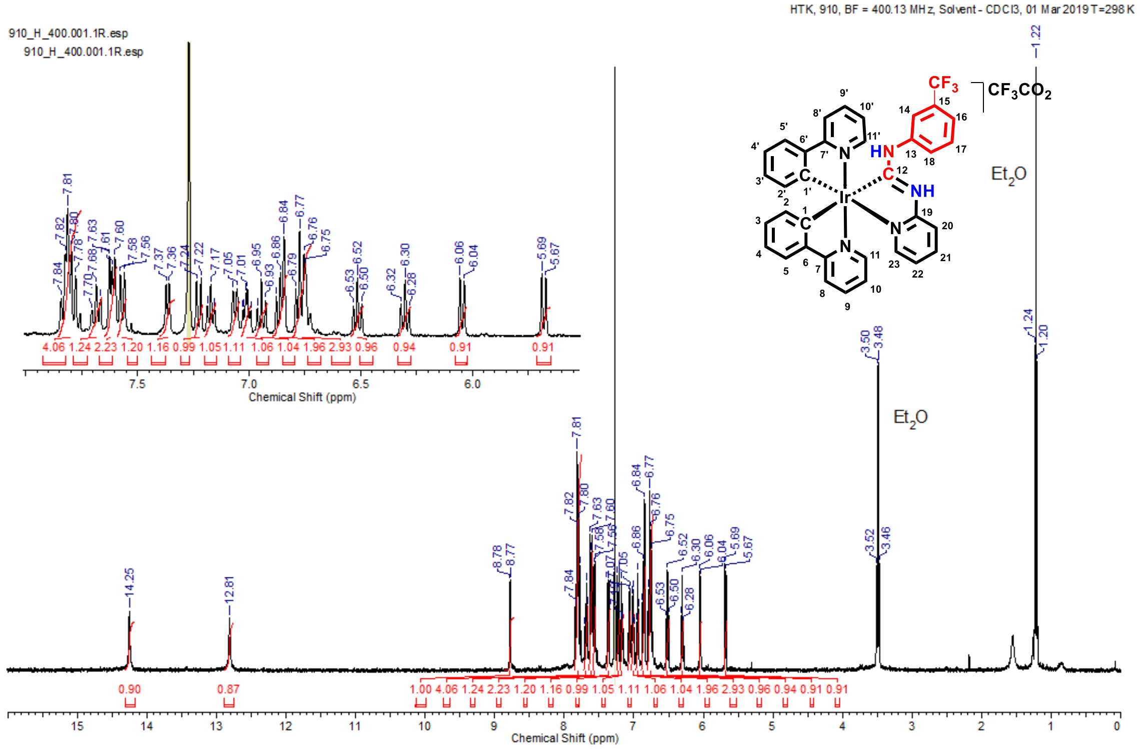

Figure S32. ^{1}H NMR spectra for **6** in $CDCl_3$.

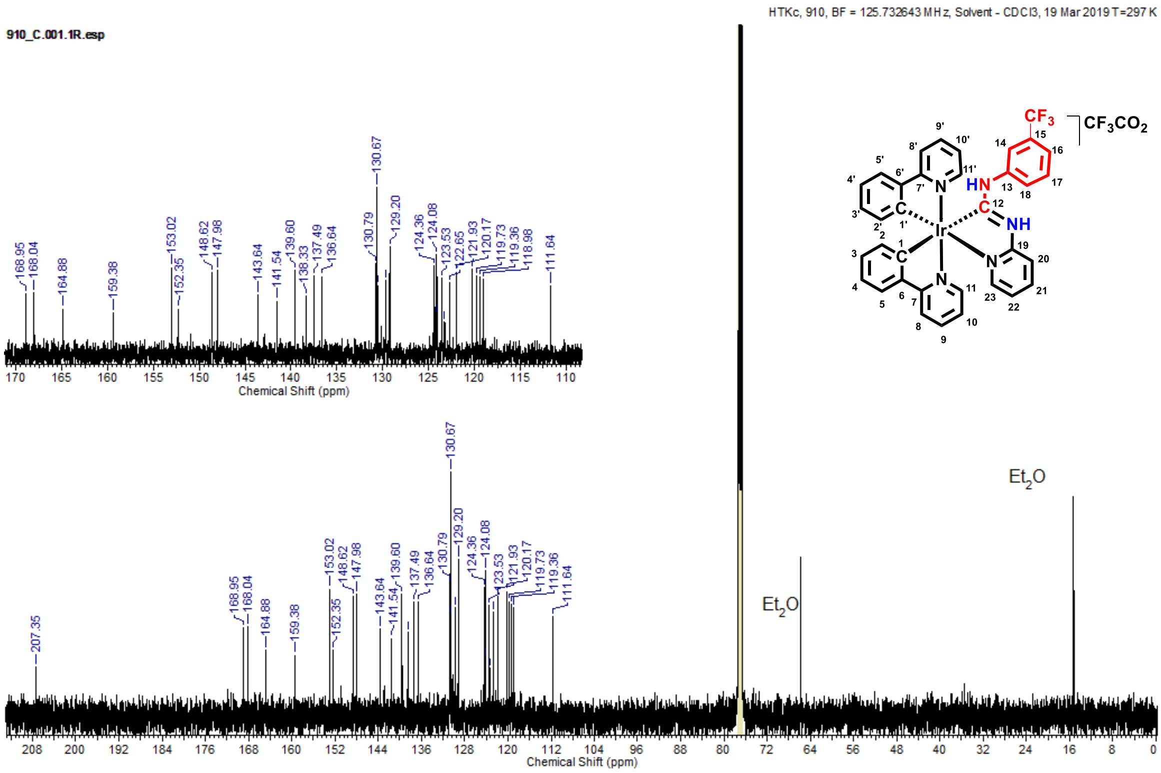

Figure S33. ^{13}C {^{1}H} NMR spectra for **6** in $CDCl_3$.

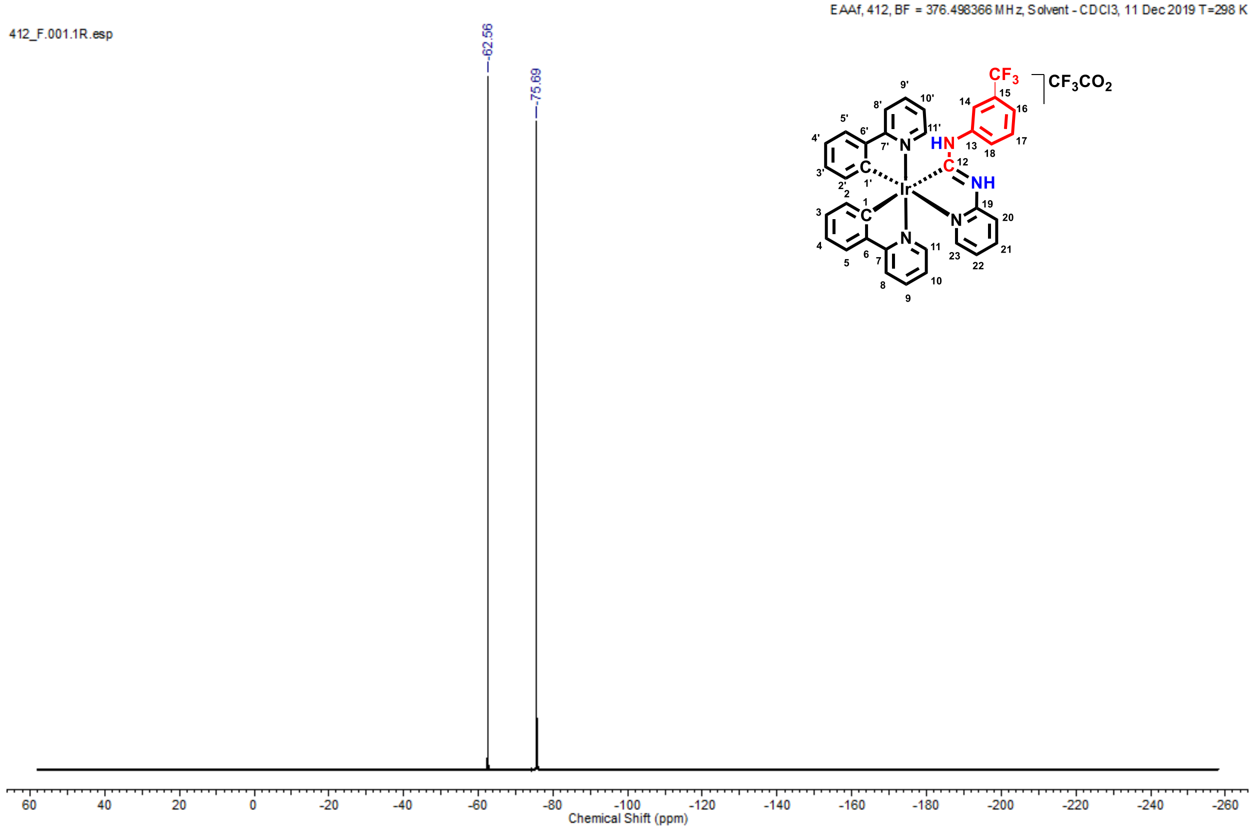

Figure S34. ^{19}F {^{1}H} NMR spectra for **6** in $CDCl_3$.

S7. FTIR spectra for complexes **3**–**6**

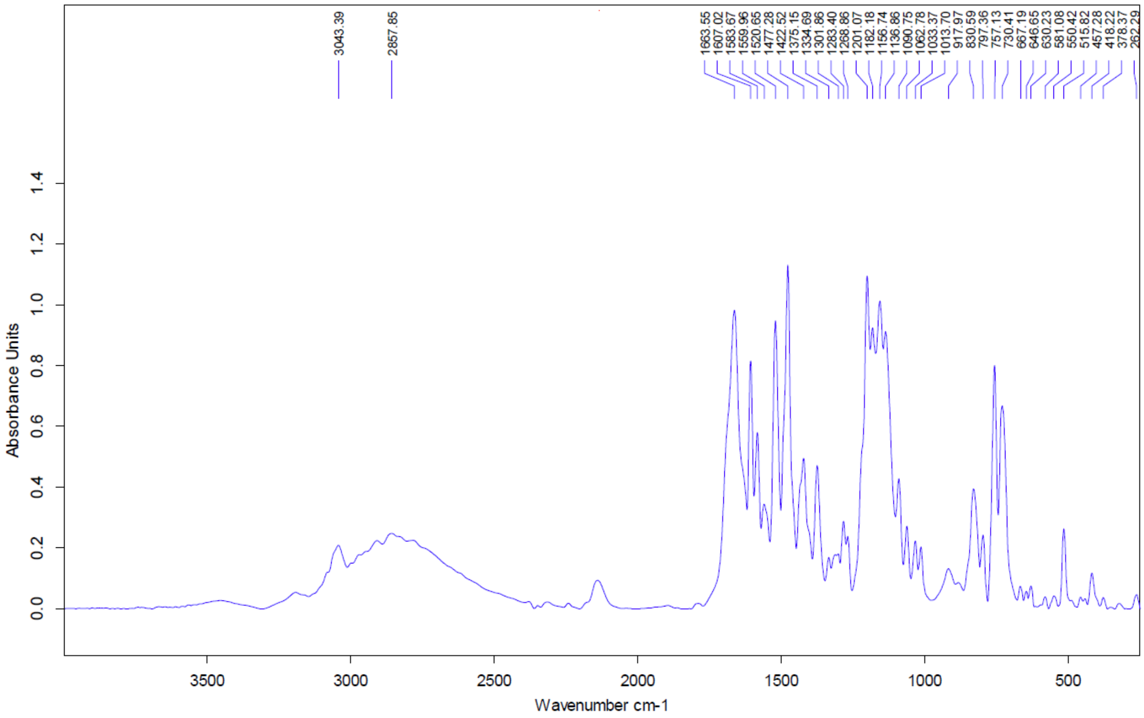

Figure S35. IR spectrum for **3** in KBr tablet.

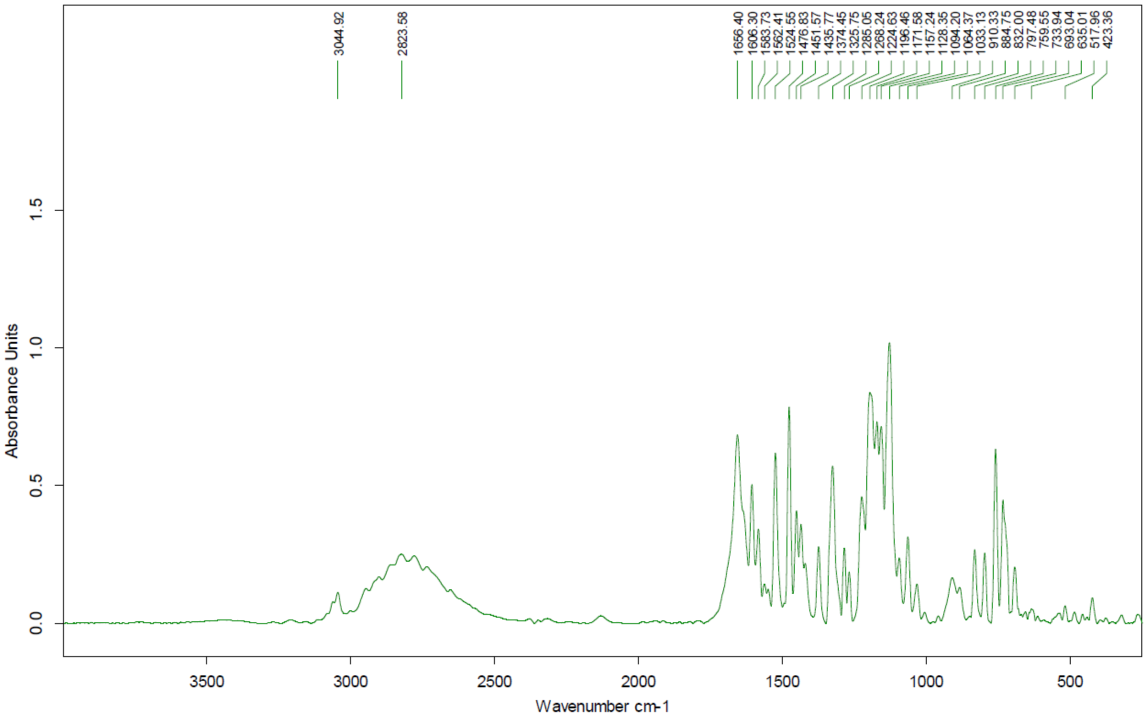

Figure S36. IR spectrum for **4** in KBr tablet.

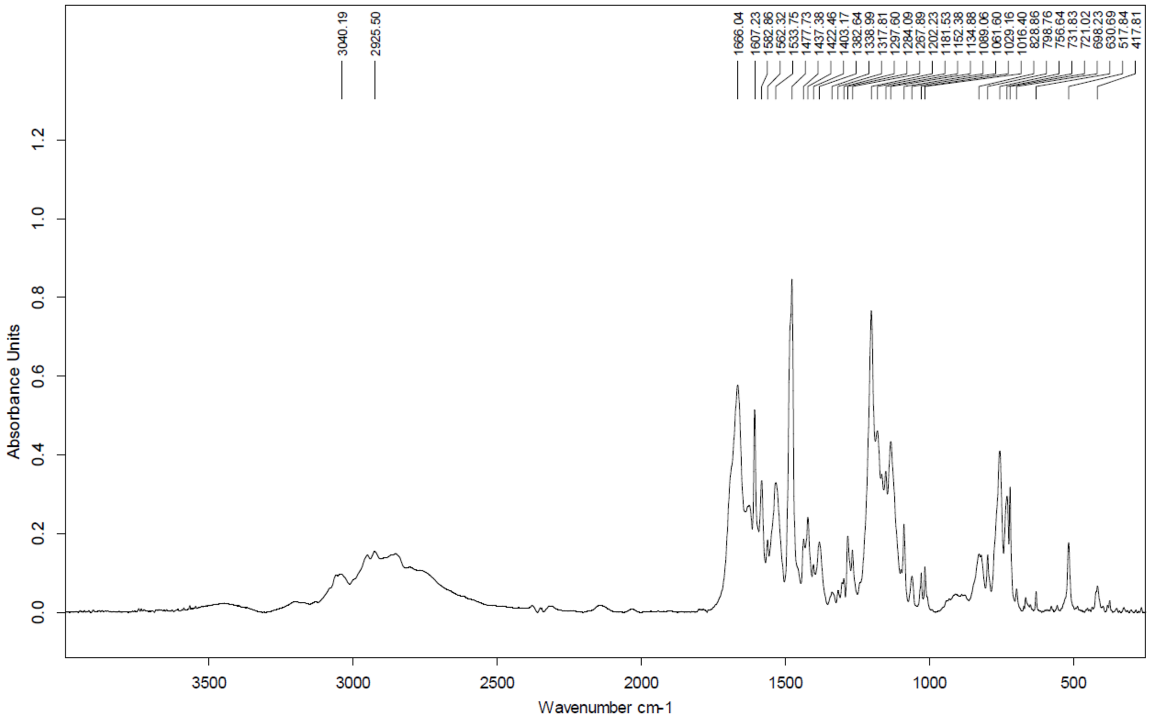

Figure S37. IR spectrum for **5** in KBr tablet.

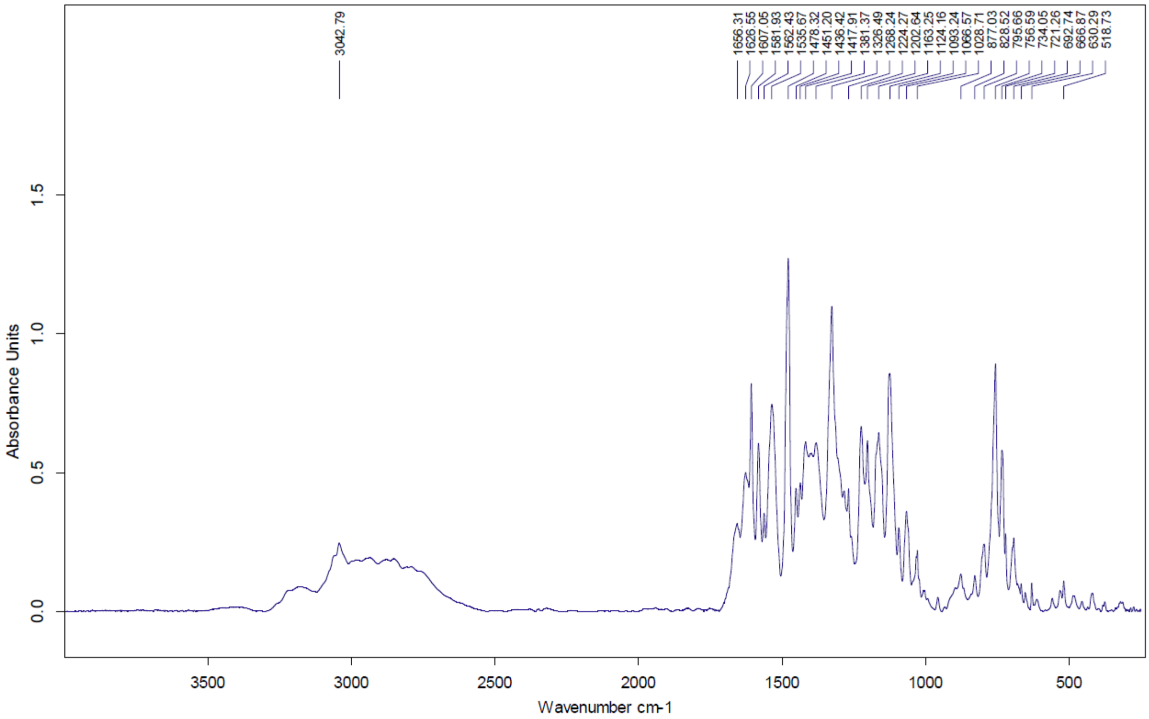

Figure S38. IR spectrum for **6** in KBr tablet.

S8. Mass spectra for complexes **3**–**6**

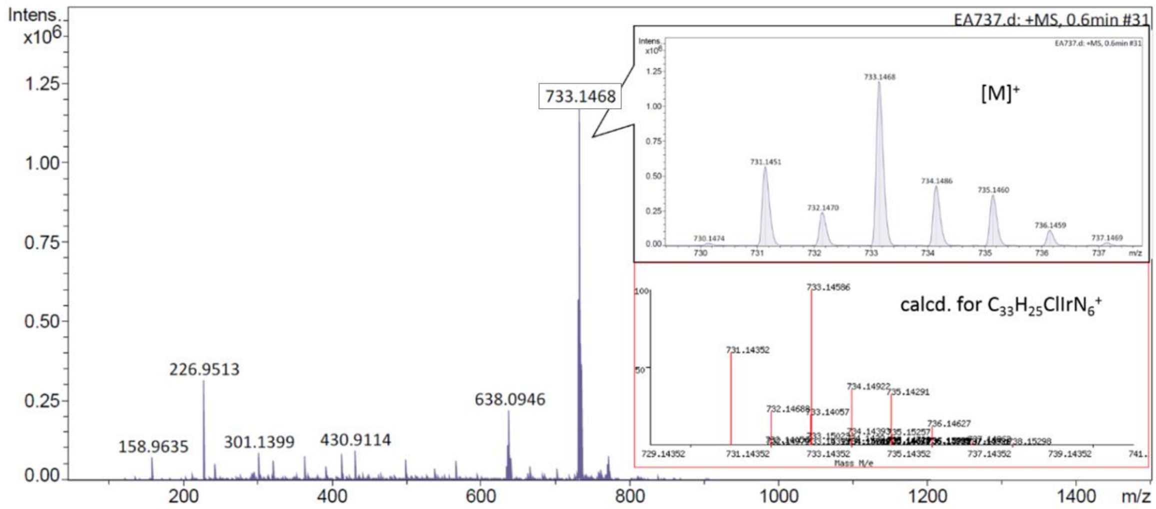

Figure S39. ESI-MS for **3**.

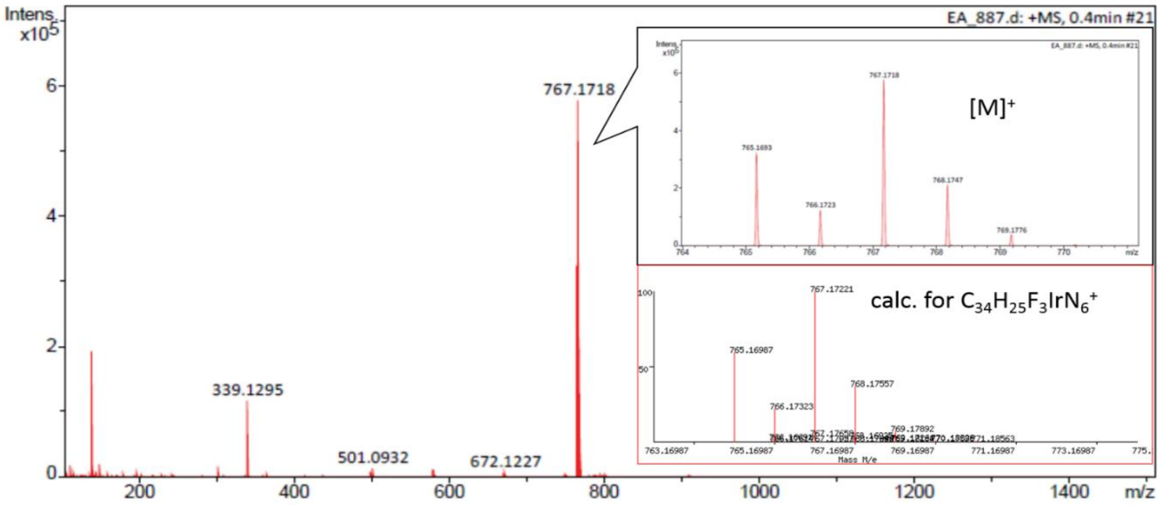

Figure S40. ESI-MS for **4**.

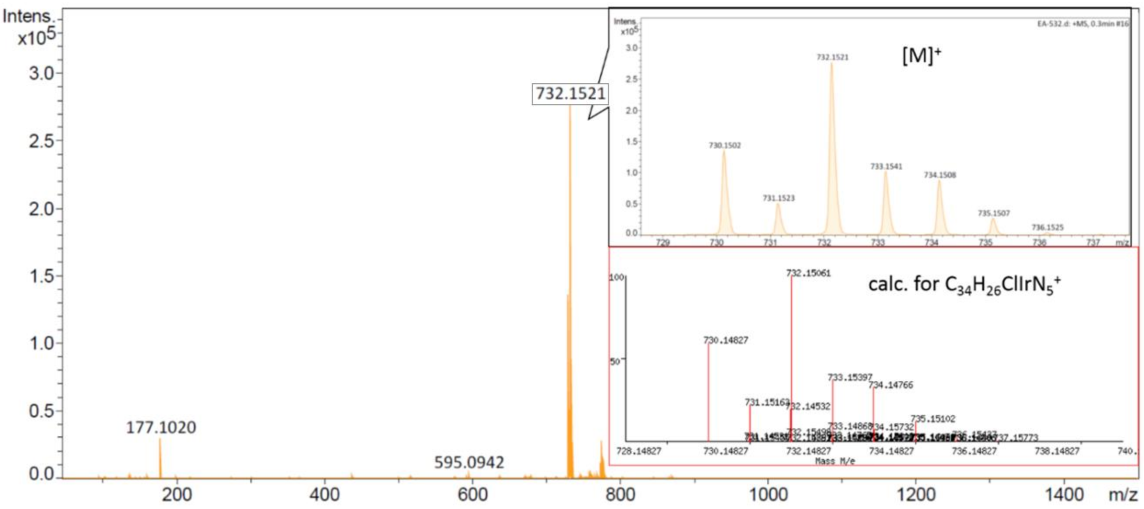

Figure S41. ESI-MS for **5**.

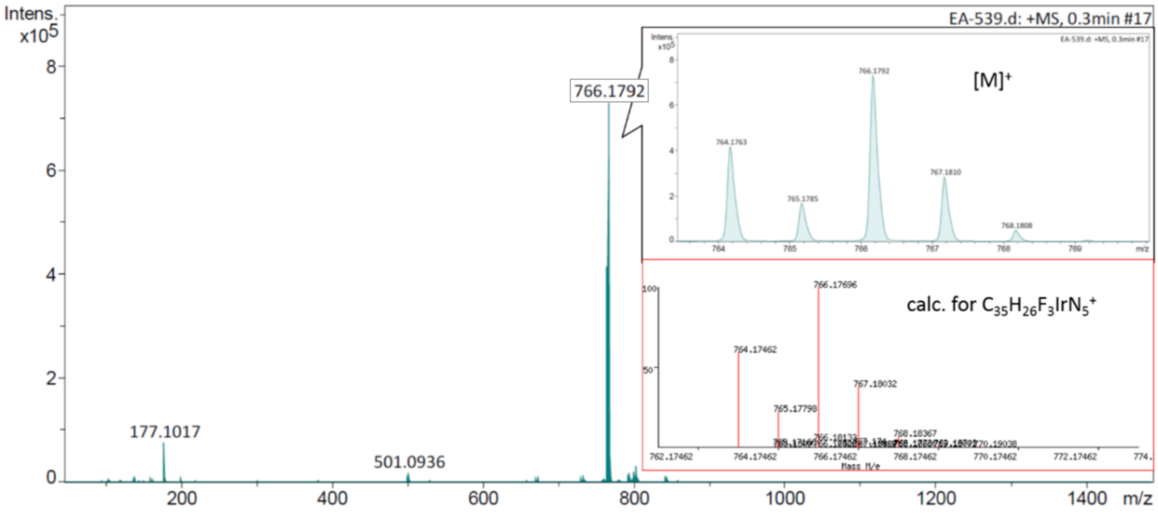

Figure S42. ESI-MS for **6**.

S9. References

1. Nonoyama, M., [Benzo[h]quinolin-10-yl-N]iridium(III) complexes. *Bull. Chem. Soc. Jap.* **1974**, *47*, 767–768.

2. Luzyanin, K. V., Pombeiro, A.J.L., Haukka, M., and Kukushkin, V.Y., Coupling between 3-Iminoisoindolin-1-ones and Complexed Isonitriles as a Metal-mediated Route to a Novel Type of Palladium and Platinum Iminocarbene Species. *Organometallics* **2008**, *27*, 5379–5389.

3. Maity, A., Le, L.Q., Zhu, Z., Bao, J., and Teets, T.S., Steric and Electronic Influence of Aryl Isocyanides on the Properties of Iridium(III) Cyclometalates. *Inorg. Chem.* **2016**, *55*, 2299–2308.

4. Origin(Pro), Version 2025; OriginLab Corporation: Northampton, MA, USA, **2025**.

5. Brouwer, A. M., Standards for photoluminescence quantum yield measurements in solution (IUPAC Technical Report). *Pure Appl. Chem.* **2011**, *83*, 2213–2228.

7. Dolomanov, O. V.; Bourhis, L. J.; Gildea, R. J.; Howard, J. A. K.; Puschmann, H., OLEX2: a complete structure solution, refinement and analysis program. *J. Appl. Crystallogr.* **2008,** *42*, 339–341.

8. Agilent, C. *CrysAlis PRO*, Agilent Technologies Ltd: Yarnton; Oxfordshire, **2014**.

9. Kohn, W.; Becke, A. D.; Parr, R. G., Density Functional Theory of Electronic Structure. *J. Phys. Chem.* **1996**, *100*, 12974–12980.

10. Lee, C.; Yang, W.; Parr, R. G., Development of the Colle-Salvetti correlation-energy formula into a functional of the electron density. *Phys. Rev. B* **1988**, *37*, 785–789.

11. Becke, A. D., Density-functional exchange-energy approximation with correct asymptotic behavior. *Phys. Rev. A* **1988**, *38*, 3098–3100.

12. Weigend, F.; Ahlrichs, R., Balanced basis sets of split valence, triple zeta valence and quadruple zeta valence quality for H to Rn: Design and assessment of accuracy. *Phys. Chem. Chem. Phys.* **2005,** *7*, 3297–3305.

13. Neese, F., The ORCA program system. *Wiley Interdiscip. Rev. Comput. Mol. Sci.* **2012,** *2*, 73–-78.

14. Zhurko, G. A. *Chemcraft - graphical software for visualization of quantum chemistry computations*, Version 1.8.

15. Kinzhalov, M. A., Boyarskiy, V.P., Luzyanin, K.V., Dolgushin, F.M., and Kukushkin, V.Y., Metal-mediated coupling of a coordinated isocyanide and indazoles. *Dalton Trans* **2013**, *42*, 10394–10397.

16. Kinzhalov, M. A.; Eremina, A. A.; Smirnov, A. S.; Suslonov, V. V.; Kukushkin, V. Y.; Luzyanin, K. V., Cleavage of acyclic diaminocarbene ligands at an iridium(III) center. Recognition of a new reactivity mode for carbene ligands. *Dalton Trans.* **2019**, *48*, 7571–7582.

17. Eremina, A. A.; Kinzhalov, M. A.; Katlenok, E. A.; Smirnov, A. S.; Andrusenko, E. V.; Pidko, E. A.; Suslonov, V. V.; Luzyanin, K. V., Phosphorescent Iridium(III) Complexes with Acyclic Diaminocarbene Ligands as Chemosensors for Mercury. *Inorg. Chem.* **2020**, *59*, 2209–2222.

18. Allen, F. H.; Kennard, O.; Watson, D. G.; Brammer, L.; Orpen, A. G.; Taylor, R., Tables of bond lengths determined by X-ray and neutron diffraction. Part 1. Bond lengths in organic compounds. *J. Chem. Soc., Perkin Trans. 2* **1987**, *0* (12), S1–S1.
